# CO_2_ silylation at a tucked-in iron(II) complex

**DOI:** 10.3762/bjoc.22.104

**Published:** 2026-09-23

**Authors:** Logan J Taylor, Marcus W Drover

**Affiliations:** 1 Department of Chemistry, Western University, 1151 Richmond Street, London, ON, N8K 3G6, Canada

**Keywords:** carbon dioxide, iron, organometallics, silylation, tucked-in complex

## Abstract

Carbon dioxide (CO_2_) capture and valorization remain central challenges in sustainable chemistry due to the persistence of CO_2_ as a major greenhouse gas. Herein, we describe the functionalization of a CO_2_-activated organometallic complex, [(Cp*–CO_2_)Fe(diphosphine)] (**1**; Cp* = C_5_Me_5_) in which CO_2_ is selectively incorporated into the Cp* ligand framework. Treatment of **1** with trimethylsilyl chloride (TMSCl) affords [{Cp*–C(O)–O–TMS}Fe(diphosphine)Cl] (**2**) via silylation of the Fe–O(CO_2_) linkage. Chloride abstraction using NaBPh_4_ generates the putative complex [{Cp*–C(O)–O–TMS}Fe(diphosphine)]^+^ (**3**), featuring a labile dative R_2_C=O→Fe interaction that is readily displaced by acetonitrile to give [{Cp*–C(O)–O–TMS}Fe(diphosphine)(NCCH_3_)]^+^ (**4**). Reduction of the ester functionality in **2** using HBCy_2_ (Cy = cyclohexyl) provides access to new borylated complexes. Collectively, these results demonstrate the cooperative capture and stepwise CO_2_ functionalization at a common Cp* ligand framework.

## Introduction

Carbon dioxide (CO_2_) is a prominent greenhouse gas generated by many natural pathways, including animal respiration and plant death [1], but also increasingly through industrial processes such as the burning of fossil fuels [2]. Greenhouse gases are characterized by their inability to escape Earth’s atmosphere, which in turn prevents the escape of heat – a key contributor to climate change [3]. Fossil fuel-derived CO_2_ emissions continue to increase, with approximately 43% of annual CO_2_ emissions remaining in the atmosphere; this has led to a growing interest in CO_2_ remediation strategies [2]. Organometallic compounds are well-known for their use in CO_2_ activation, with examples spanning the s- [4–5], p- [6], d- [7–8], and f-blocks [9]. For d-block elements, examples include early [9] to late transition metals [10], with ongoing efforts focused on optimizing CO_2_ conversion catalysis [11–12] into useful products, such as CO, formaldehyde, methanol, and more [6,9–10].

**Scheme 1:**
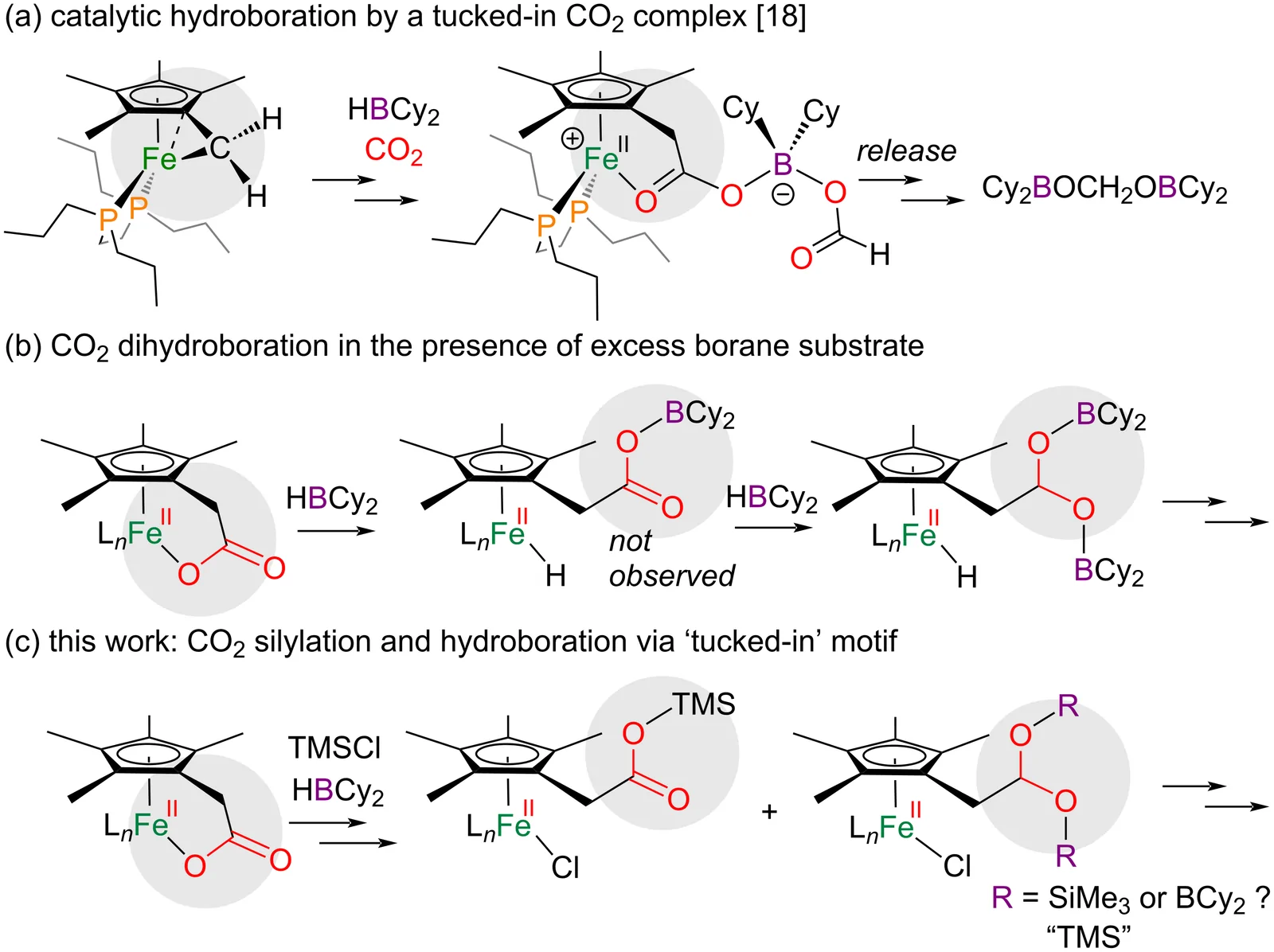
(a) Catalytic hydroboration by a tucked-in CO_2_ complex ([18]); (b) CO_2_ dihydroboration in the presence of excess borane substrate; (c) this work: CO_2_ silylation and hydroboration via ‘tucked-in’ motif.

Of reduction strategies, examples of CO_2_ hydroboration and hydrosilylation to give bisboryl or silyl ethers have been achieved, which once hydrolyzed provide access to C1-containing synthons. CO_2_ hydroboration has been demonstrated using a wide range of metals including manganese [13], nickel [14], and palladium [14] to generate borylated analogues of methanol, with various reagents such as pinacolborane (HBPin) [13] and 9-borabicyclo[3.3.1]nonane (9-BBN) [13–14]. Metal-free examples using p-block elements such as silicon [15] and nitrogen [16] have also been shown. Recently, our group contributed to this space, reporting a tucked-in iron complex that reacts with CO_2_ to give a ferralactone (Scheme 1a and b) [17] which undergoes addition of dicyclohexylborane (HBCy_2_) to give [(Cp*–BCy**_2_**)Fe(diphosphine)H] – a competent catalyst for CO_2_ dihydroboration using HBCy_2_ [18]. We suggest this reaction occurs via a CO_2_ B–O templating strategy using a pendent secondary coordination sphere (SCS) borane moiety [18].

CO_2_ (hydro)silylation is complementary to hydroboration, leading to the formation of robust Si–O bonds. Indeed, Leitner and co-workers demonstrated that a cobalt–triazine complex can catalytically produce silylated analogues of formate, methanol, and formaldehyde in the presence of silane, with product selectivity depending on the reaction temperature [19]. Iron has also been shown to hydrosilylate CO_2_ by Cantat and co-workers, who used different iron(II) phosphine salts to generate formamides and methylamines in the presence of different amines [20]. Other contributions using cobalt pincer complexes [21], NHC-stabilized (NHC = N-heterocyclic carbene) stannyliumylidene complexes [22], and oxorhenium tridentate PNN catalysts [23] to reduce CO_2_ have also been established. Relevant to this work, a 2022 report by Horáček demonstrated the silylation of CO_2_ using a titanium ‘tucked-in’ compound in combination with trimethylsilyl chloride (TMSCl) [24]. Building on these precedents, we sought insight into the reactivity of [(Cp*–CO_2_)Fe(diphosphine)] with a silyl electrophile. The unusual CO_2_-functionalized Cp*Fe framework provides a rare platform for interrogating how tucked-in motifs participate in reductive transformations with silanes. These studies complement our prior work on hydroboration (Scheme 1b and c) and aim to elucidate broader patterns in base-metal-mediated CO_2_ reduction chemistry.

## Results and Discussion

**Scheme 2:**
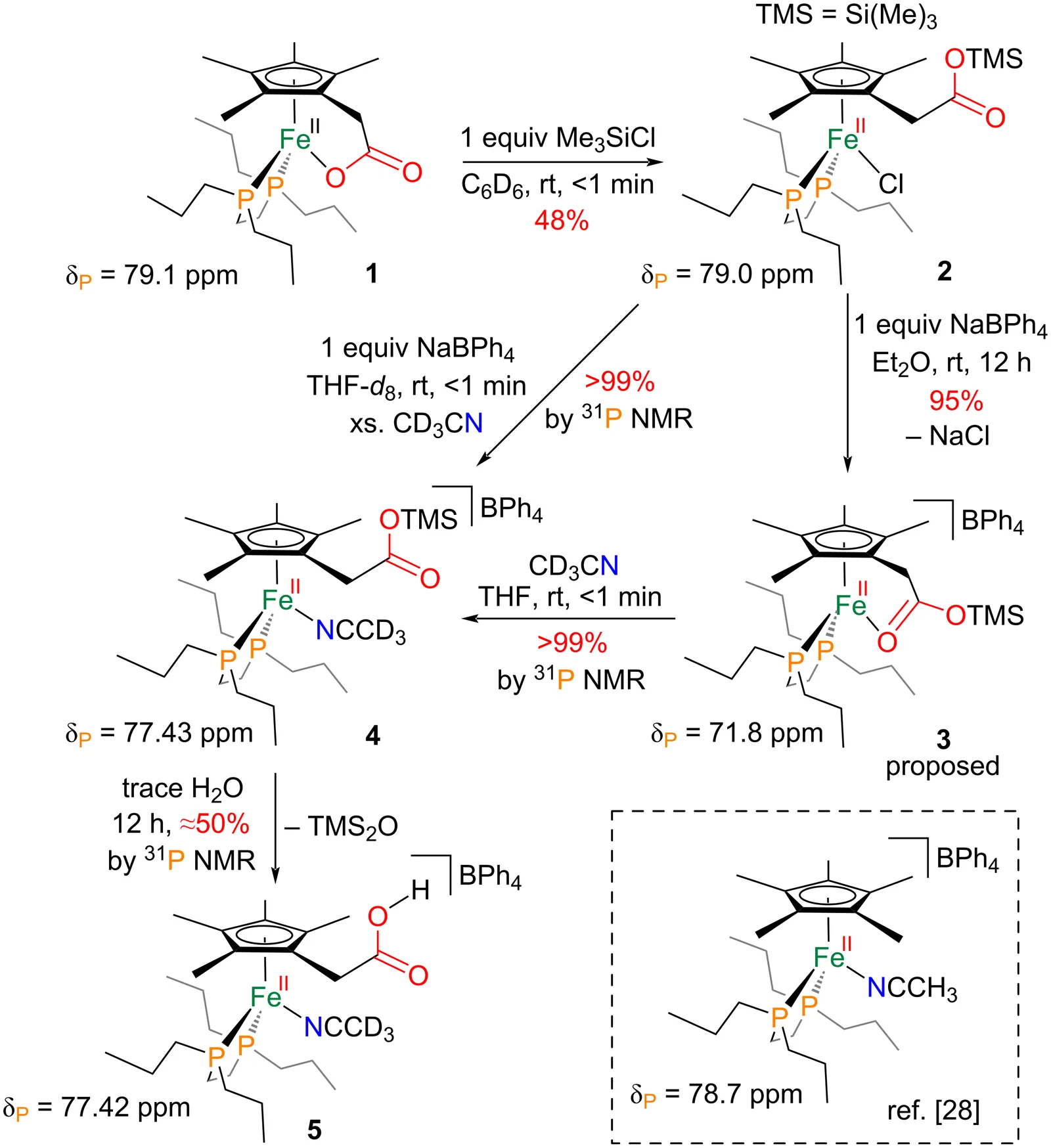
Synthesis of the silylated CO_2_ adduct **2** and subsequent chloride abstraction.

**Figure 1:**
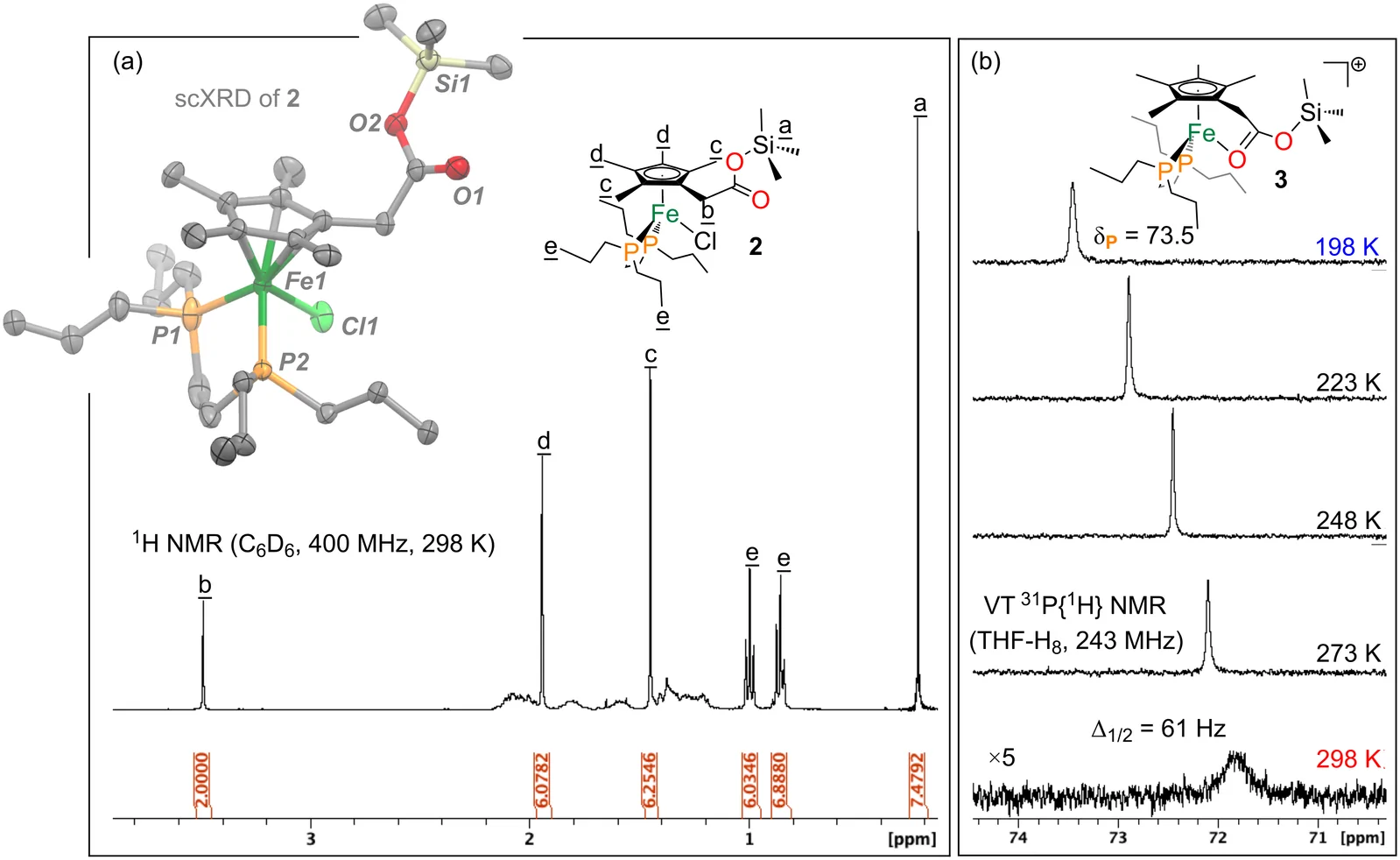
(a) **2**, ^1^H NMR, C_6_D_6_, 400 MHz, 298 K. Inset shows the molecular structure of **2** with ellipsoids drawn at 50% probability. Hydrogen atoms are omitted for clarity; (b) variable-temperature (VT) ^31^P{^1^H} NMR for **3** from 298 to 198 K (THF-H_8_, 243 MHz). Broad signals in ^1^H NMR baseline of **2** attributed to d*n*ppe CH_2_ signals.

To begin, [(Cp*–CO_2_)Fe(d*n*ppe)] (**1**) [17] was prepared by reaction of the tucked-in complex, [(η^6^–C_5_Me_4_–CH_2_)Fe^II^(d*n*ppe)] with 1 atm of CO_2_ at room temperature. Complex **1** has a characteristic ^31^P NMR spectroscopic signature at δ_P_ = 79.1 ppm and a distinct ν(CO) stretch at 1622 cm^−1^. Treatment of **1** with 1 equiv of trimethylsilyl chloride (TMSCl) yielded a slightly darker purple solution, with a very similar ^31^P{^1^H} NMR shift to **1** at δ_P_ = 79.0 ppm, which mirrors the value observed for the known complex [Cp*Fe(d*n*ppe)Cl] (δ_P_ = 79.4 ppm) (Scheme 2) [25]. Of note, both complex **1** and its tucked-in precursor show no productive reactivity with hydridosilanes such as Ph_3_SiH. By ^1^H,^29^Si HMBC NMR spectroscopy, TMS incorporation was corroborated by the presence of a cross-signal at δ_H_ = 0.22 ppm (9H) and δ_Si_ = −24 ppm, indicating formation of [{Cp*–C(O)–O–TMS}Fe(d*n*ppe)Cl] (**2**), having a peripherally silylated CO_2_ group. Further examination of the ^1^H NMR spectra confirms *C_s_* symmetry, rendering the functionalized CH_2_ protons equivalent (δ_H_ = 3.49 ppm) (Figure 1a). By LIFDI–MS, **2** displays a molecular ion at *m/z* = 604.2474 (calcd. *m/z* = 604.2485 for C_28_H_55_O_2_ClFeP_2_Si). The structure of **2** was further confirmed by scXRD using purple crystals grown from a saturated pentane solution at −35 °C. The solid-state structure reveals an Fe–Cl bond length of 2.353(1) Å and a Si–O bond length of 1.699(2) Å (Figure 1a, inset). The silylated ester fragment exhibits bond metrics of r(C–C(O)) = 1.509(3) Å, r(C=O) = 1.207(2) Å, and r(C–O) = 1.346(3) Å. The lengths of the C–O and C=O bonds fall within the expected literature range for ester functional groups of 1.35–1.40 Å and 1.1–1.2 Å, respectively [26].

Motivated by the clean silylation reactivity of **1**, we explored whether removal of the iron-bound Cl ligand in **2** could promote onwards reactivity, potentially enabling interaction between the Fe center and the C=O group. As such, **2** was reacted with sodium tetraphenylborate (NaBPh_4_) in diethyl ether over 12 hours, affording a cloudy yellow solution. Subsequent analysis by ^31^P{^1^H} NMR spectroscopy revealed a broad peak at δ_P_ = 71.8 ppm (Δ_1/2_ = 60 Hz), suggestive of abstraction (Figure 1b). The width of this resonance suggested an equilibrium involving [{Cp*–C(O)–O–TMS}Fe(d*n*ppe)]^+^, potentially between a carbonyl oxygen-bound and unbound form, or between the bound species and its dinitrogen adduct [{Cp*–C(O)–O–TMS}Fe(d*n*ppe)(N_2_)]^+^ (δ_P_ = 71.2 ppm for [Cp*Fe(d*n*ppe)(N_2_)]^+^, for example). The unbound (N_2_-free) species adopts a triplet electronic configuration (*S* = 1) and is higher in energy than its corresponding N_2_ adduct (*S* = 0) [27]. These possibilities prompted further investigation by variable-temperature NMR spectroscopy (Figure 1b). Upon cooling to −75 °C, this ^31^P NMR peak significantly sharpens and moves to δ_P_ = 73.5 ppm, which is attributed to the coordination equilibrium, as described above. Further supporting an “on–off” equilibrium in **3**, introduction of the weak L–type donor, acetonitrile, provided a ^31^P NMR resonance at δ_P_ = 77.4 ppm; this is similar to the model complex, [Cp*Fe(d*n*ppe)NCCH_3_]**^+^** (δ_P_ = 78.7 ppm) [28], suggesting formation of [{Cp*–C(O)–O–TMS}Fe(d*n*ppe)NCCH_3_]^+^ (**4**) and displacement of the weak R_2_C=O**→**Fe interaction (Scheme 2). During efforts to characterize **4**, desilylation occurred, likely due to the presence of trace amounts of moisture in the THF or MeCN used, giving **5** (δ_H_ = 11.1 ppm) and 0.5 equiv TMS_2_O (δ_H_ = 0.07 ppm) [29] (see Supporting Information File 1). By ESI–MS, **5** displays an [M − H]^+^ signal at *m*/*z* = 496.2309 (calcd. *m*/*z* = 496.2322 for C_25_H_46_FeO_2_P_2_); a signal for **4** was not observed, again highlighting the ease by which the –OTMS group is cleaved.

**Scheme 3:**
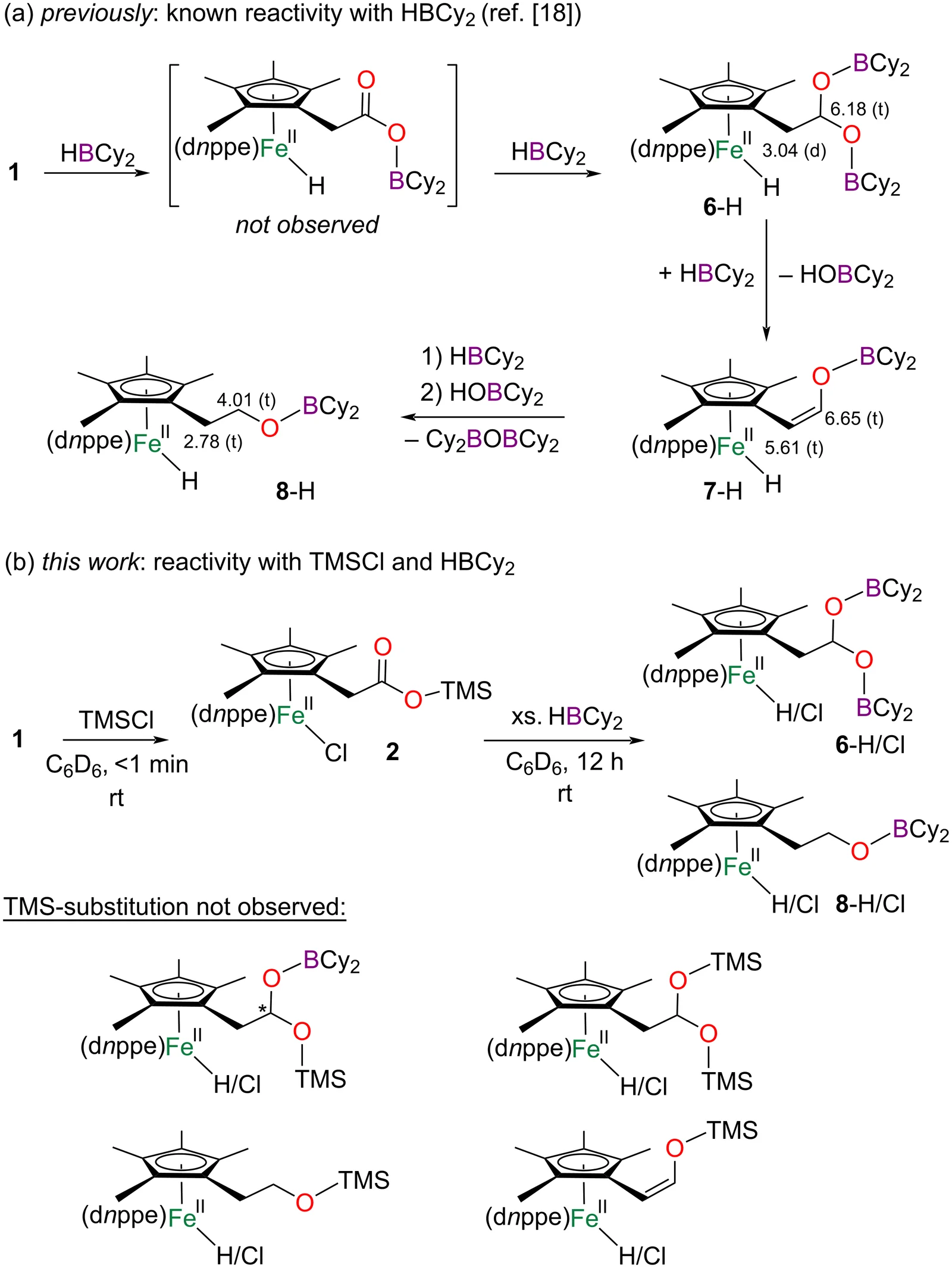
Observed reactivity between **1**, TMSCl, and HBCy_2_. Numbers refer to ^1^H NMR shifts (multiplicity).

Like **1**, complex **2** also undergoes reduction upon treatment with excess HBCy_2_. Multinuclear NMR spectroscopic analysis supports the formation of several C_2_-functionalized Cp* compounds, including the bisborylacetal (**6**–H), vinyloxyborane (**7**–H), and ethoxyborane (**8**–H) complexes where X–H refers to an {Fe–H} species; these were identified in the crude reaction mixture based on characteristic NMR signatures (Scheme 3) and from our previous work independently reacting **1** with HBCy_2_ [18]. These products arise from successive borane additions to the CO_2_-derived C_2_ unit, ultimately leading to the formation of ethoxyborane complex **8**–H. Unlike **1**, the use of the {Fe–Cl} precursor **2** further complicates analysis of the product mixture, possessing several {Fe–Cl}-containing species (X–Cl) in addition to {Fe–H}-containing complexes **6**–H and **8**–H. By LIFDI–MS of the product mixture, two predominant signals are seen at *m*/*z* = 660.4416 (calcd. 660.4423 for C_37_H_71_OBFeP_2_ for **8**–H) and *m*/*z* = 694.4025 (calcd. 694.4033 for C_37_H_70_OBClFeP_2_ for **8**–Cl). Consistent with the strong leaving ability of –OTMS, silylated Cp* complexes were not observed by crude ^1^H NMR spectroscopy nor LIFDI–MS. The fate of –SiMe_3_ was not determined, however, TMS signals could be observed between 0.1–0.3 ppm via ^1^H NMR spectroscopy. Formation of Me_3_SiH during the synthesis of **6**–H/Cl is possible, however, it is highly volatile (bp 6.7 °C) [30], and a characteristic multiplet (δ_Si–H_ ≈ 4.0 ppm) [31] was not observed by ^1^H NMR spectroscopy.

Overall, this reductive chemistry provides a route to Cp* products in which CO_2_ serves as a C1 synthon at a silylated iron complex, enabling access to reduced ethyl-functionalized derivatives, albeit with lower selectivity for iron hydride products when compared to previous results using hydroboration of **1** [18].

## Conclusion

In summary, we have demonstrated that a CO_2_-functionalized tucked-in iron complex can be stepwise derivatized. Electrophilic silylation with TMSCl functionalizes the Fe–CO_2_ linkage to afford a silyl ester. Subsequent chloride abstraction suggests generation of a cationic complex featuring a weak, fluxional intramolecular carbonyl O→Fe interaction that is readily displaced by the neutral donor ligand, acetonitrile, highlighting the hemilabile nature of the tethered ester functionality. Preliminary hydroboration studies further demonstrate that the silylated CO_2_ fragment remains susceptible to downstream desilylation and reduction. Collectively, these results provide new opportunities for the development of organometallic strategies that combine CO_2_ activation with selective C_1_–derivatization.

## Experimental

### General considerations

All experiments were carried out employing standard Schlenk techniques under an atmosphere of dry nitrogen employing degassed, dried solvents. Benzene and THF were tested with a standard purple solution of sodium benzophenone ketyl in tetrahydrofuran to confirm effective moisture removal. Acetonitrile was dried over molecular sieves and degassed by three freeze–pump–thaw cycles [32]. All other reagents were purchased from commercial vendors and used without further purification unless otherwise stated. [(Cp*–CO_2_)Fe(d*n*ppe)] (**1**) was prepared according to literature procedures [17].

### Physical methods

All NMR data were recorded with a Bruker AVIII HD 400 MHz, a Bruker Neo 600 MHz, or a Varian INOVA 600 instrument. ^1^H NMR spectra are reported in parts per million (ppm) and are referenced to residual solvent, e.g., ^1^H (C_6_D_6_): δ = 7.16; ^13^C (C_6_D_6_): δ = 128.06; coupling constants are reported in Hz. ^11^B, ^13^C, ^29^Si, and ^31^P NMR spectra were performed as proton-decoupled experiments (unless explicitly stated otherwise) and are reported in ppm. Selective irradiation experiments (^1^H 1D TOCSY) were performed by selective irradiating signal of interest using a Bruker Neo 600 MHz instrument. 2D NMR experiments (^1^H,^1^H COSY, ^1^H,^13^C HSQC) were run using standard acquisition parameters on a Bruker Neo 600 MHz instrument. ^1^H,^29^Si HSQC was performed with a Bruker AVIII HD 400 MHz instrument. VT ^31^P{^1^H} NMR was performed with a Varian INOVA 600 instrument. Mass spectrometry was carried out with a Thermo Scientific Orbitrap Exploris 120 Instrument.

### Preparation of compounds

[(η^5^–C_5_Me_4_–CH_2_–CO_2_Si(CH_3_)_3_)Fe(d*n*ppe)Cl] (**2**; C_28_H_55_FeO_2_P_2_ClSi, MW = 604.6 g/mol): In a glovebox, **1** (23 mg, 0.046 mmol) was dissolved in benzene (2 mL) in a 20 mL scintillation vial. Trimethylsilyl chloride (5.89 μL, 0.046 mmol) was added and the reaction occurred immediately. The mixture was then washed three times with pentane (3 × 2 mL) and filtered through Celite^®^ 545. Removal of volatiles in vacuo gave **2** as a purple solid (11.4 mg, 48%).^1^H NMR (400 MHz, C_6_D_6_, 298 K) δ_H_ 3.49 (s, 2H, η^5^-C_5_Me_4_-(C*H*_2_)), 2.09 (m, 6H, overlapping C*H*_2_ signals), 1.94 (s, 6H, η^5^-C_5_*Me*_4_-(µ-C*H*_2_)), 1.83 (m, 2H, C*H*_2_), 1.59 (m, 2H, C*H*_2_), 1.45 (s, 6H, η^5^-C_5_*Me*_4_-(µ-CH_2_)), 1.30 (m, 10H, C*H*_2_ signals), 0.99 (t, *J*_H,H_ = 7.2 Hz, 6H, P-CH_2_-CH_2_-C*H*_3_) 0.85 (t, *J*_H,H_ = 7.2 Hz, 6H, P-CH_2_-CH_2_-C*H*_3_), 0.22 (s, 9H, Si-(C*H*_3_)_3_); ^31^P{^1^H} NMR (162 MHz, C_6_D_6_, 298 K) δ_P_ 79.0 ppm; ^1^H,^29^Si NMR HMBC (400 MHz, C_6_D_6_, 298 K) δ_Si_ −24 ppm; ^13^C{^1^H} NMR (101.5 MHz, C_6_D_6_, 298 K) δ_C_ 171.7 (s, C_5_Me_4_-CH_2_-*C*O_2_), 86.1 (s, *C*_5_Me_4_-CH_2_-CO_2_), 79.9 (s, *C*_5_Me_4_-CH_2_-CO_2_), 79.1 (s, *C*_5_Me_4_-CH_2_-CO_2_), 34.6 (s, *C*H_2_), 31.7 (m, *C*H_2_), 29.2 (m, *C*H_2_), 23.2 (m, *C*H_2_), 19.3 (s, *C*H_2_), 18.4 (s, *C*H_2_), 16.6 (m, *C*H_2_), 11.5 (s, *C*H_3_), 11.2 (s, *C*H_3_), −0.24 (s, Si-(*C*H_3_)_3_; FTIR (ATR, benzene film) ν: 1,716 cm^−1^ (C=O); HRMS–LIFDI (*m*/*z*): [M]^+^ calcd for C_28_H_55_FeO_2_P_2_ClSi, 604.2485; found, 604.2474.

[(η^5^–C_5_Me_4_–CH_2_–CO_2_Si(CH_3_)_3_)Fe(d*n*ppe)][BPh_4_] (**3**; C_52_H_72_FeO_2_P_2_SiB, MW = 888.9 g/mol): In a glovebox, compound **2** (11.4 mg, 0.019 mmol) was dissolved in diethyl ether in a 20 mL scintillation vial. NaBPh_4_ (6.1 mg, 0.018 mmol, 0.95 equiv) was added and the reaction mixture was stirred for 24 h. Volatiles were removed in vacuo, and the solids washed with pentane (3 × 2 mL), where the residue was then dissolved in THF, filtered through Celite^®^ 545, and volatiles removed in vacuo to afford **3** as a yellow–beige solid (18.3 mg, >99%). ^31^P{^1^H} NMR (243 MHz, THF-H_8_, 298 K) δ_P_ 71.8 ppm (br).

[(η^5^–C_5_Me_4_–CH_2_–CO_2_Si(CH_3_)_3_)Fe(d*n*ppe)NCCD_3_][BPh_4_] (**4**; C_54_H_78_FeNO_2_P_2_SiB, MW = 929.9 g/mol): In a glovebox, compound **2** (10.6 mg, 0.018 mmol) was placed in a scintillation vial and dissolved in THF–*d*_8_. NaBPh_4_ (6 mg, 0.018 mmol) was then added, followed by the addition of approximately 5 μL of CD_3_CN, which immediately generated an orange-colored solution. Upon standing for 12 h, compound **4** slowly converted to compound **5**, in which the TMS group had been removed to reveal a carboxylic acid. This transformation was supported by the appearance of a new ^1^H NMR resonance at δ_H_ 11.1 ppm (RCO_2_H), together with a new broadened ^31^P NMR resonance at δ_P_ 77.42 ppm, slightly upfield of that observed for **4** (δ_P_ 77.43 ppm). ^1^H NMR (400 MHz, THF-*d*_8_, 298 K) δ_H_ 7.28 (m, 8H, o-C_6_H_5_ BPh_4_^−^), 6.85 (m, 8H, *m*-C_6_H_5_ BPh_4_^−^), 6.71 (m, 4H, *p*-C_6_H_5_ BPh_4_^−^), 2.98 (s, 2H, η^5^-C_5_Me_4_-(C*H*_2_)), 2.07 (m, 4H, overlapping C*H*_2_ signals), 1.86 (m, 6H, overlapping C*H*_2_ signals), 1.67 (s, 6H, η^5^-C_5_*Me*_4_-(µ-CH_2_)), 1.62 (s, 6H, η^5^-C_5_*Me*_4_-(µ-CH_2_)), 1.50 (m, 6H, overlapping C*H*_2_ signals), 1.23 (m, 4H, overlapping C*H*_2_ signals), 1.07 (m, 12H, P-CH_2_-CH_2_-C*H*_3_), 0.23 (s, 9H, Si-(C*H*_3_)_3_); ^31^P{^1^H} NMR (162 MHz, THF-*d*_8_, 298 K) δ_P_ 77.43 (s, **4**), 77.42 (br, **5**); ^11^B{^1^H} NMR (128 MHz, THF-*d*_8_, 298 K) δ_B_ −6.5 ppm; HRMS–ESI(+) (*m*/*z*): [M − H]^+^, [**5** − H]^+^ calcd for C_25_H_46_FeO_2_P_2_, 496.2322; found, 496.2309; FTIR (ATR, acetonitrile film) ν: 2,243 cm^−1^ (C≡N).

Reduction chemistry to give **6**–H/Cl and **8**–H/Cl: In the glovebox, complex **2** (8.1 mg, 0.013 mmol) was added to a 20 mL scintillation vial and dissolved in 1 mL of C_6_D_6_. HBCy_2_ (11.9 mg, 0.067 mmol) was then added and the reaction mixture was allowed to stir. Upon stirring overnight (12 h), the solution changed from purple to orange. The product mixture was dried in vacuo giving several reduced products as a yellow oil (>95% conversion of **2** based on ^31^P{^1^H} NMR spectroscopy). Due to similarity in polarity, these compounds were inseparable. Treatment of the crude reaction mixture with NaBPh_4_ (4.2 mg, 0.0123 mmol) to possibly remove any {Fe–Cl} impurities by halide abstraction in Et_2_O, followed by stirring overnight, afforded a brown solution. Removal of the solvent in vacuo, followed by washing of the resulting solid with pentane (3 × 2 mL) and filtration through Celite^®^ 545, afforded an orange–brown solution. Dissolution of this material in C_6_D_6_ resulted in minor amounts (<2 mg) of mostly two isomers having Cp*–CH_2_CH_2_OBCy_2_ connectivity, one an Fe–H (**6**–H), and the other, an Fe–Cl (**6**–Cl). ^1^H NMR (400 MHz, C_6_D_6_, 298 K, selected signals) δ_H_ 6.18 (t, ^3^*J*_H–H_ = 5.0 Hz, 1H, Cp*–CH_2_C*H*(OBCy_2_)_2_** 6**–H) [18], 5.43 (t, ^3^*J*_H–H_ = 5.0 Hz, 1H, Cp*–CH_2_C*H*(OBCy_2_)_2_** 6**–Cl), 4.01 (t, ^3^*J*_H–H_ = 7.6 Hz, 2H, Cp*–CH_2_C*H*_2_OBCy_2_** 8**–H) [18], 3.95 (t, ^3^*J*_H–H_ = 7.6 Hz, 2H, Cp*–CH_2_C*H*_2_OBCy_2_** 8**–Cl)**,** 3.04 (d, ^3^*J*_H–H_ = 5.0 Hz, 2H, Cp*–C*H*_2_CH(OBCy_2_)_2_** 6**–H), 2.89 (d, ^3^*J*_H–H_ = 5.0 Hz, 2H, Cp*–C*H*_2_CH(OBCy_2_)_2_** 6**–Cl), 2.78 (t, ^3^*J*_H–H_ = 7.6 Hz, 2H, Cp*–C*H*_2_CH_2_OBCy_2_** 8**–H)**,** 2.61 (t, ^3^*J*_H–H_ = 7.6 Hz, 2H, Cp*–C*H*_2_CH_2_OBCy_2_** 8**–Cl); ^13^C{^1^H} NMR (101 MHz, C_6_D_6_, 298 K, selected signals by ^1^H,^13^C HSQC) δ_C_ 94.42 (Cp*–CH_2_*C*H(OBCy_2_)_2_ **6**–Cl), 94.06 (Cp*–CH_2_*C*H(OBCy_2_)_2_ **6**–H), 65.8 (s; Cp*–CH_2_*C*H_2_–OBCy_2_ **8**–H), 64.18 (s; Cp*–CH**_2_***C*H**_2_**OBCy**_2_** **8**–Cl), 37.99 (Cp*–*C*H_2_CH(OBCy_2_)_2 _**6**–Cl), 36.40 (Cp*–*C*H_2_CH(OBCy_2_)_2 _**6**–H), 31.9 (s, Cp*–*C*H_2_CH_2_OBCy_2_ **8**–H), 29.72 ppm (s, Cp*–*C*H**_2_**CH**_2_**OBCy**_2_** **8**–Cl); ^11^B (128 MHz, C_6_D_6_, 298 K, selected signals) δ_B_ 45–55 ppm (br; (RO)BCy_2_); ^31^P{^1^H} NMR (162 MHz, THF-*d*_8_, 298 K) δ_P_ 97.8 (d, *J*_H,P_ = 21.0 Hz, **6**–H), 79.0 (s, **6**–Cl); HRMS–LIFDI (*m*/*z*): [M]^+^ calcd for **8**–H, C_37_H_71_BFeOP_2_, 660.4423; found, 660.4416; [M]^+^ calcd for **8**–Cl, C_37_H_70_BClFeOP_2_, 694.4033; found, 694.4025; [M]^+^ calcd for **6**–H, C_49_H_92_B_2_FeO_2_P_2_, 852.6108; found, 852.6102.

## Supporting Information

Spectroscopic data for all complexes. CCDC 2575113 contains the supplementary crystallographic data for this paper. These data can be obtained free of charge from The Cambridge Crystallographic Data Centre via https://www.ccdc.cam.ac.uk/data_request/cif.

File 1 Copies of spectra and crystallography details.

File 2 Crystallographic information file (CIF) and checkCIF file.

## Data Availability

All data that supports the findings of this study is available in the published article and/or the supporting information of this article.
