# Supplementary material for: CO2 silylation at a tucked-in iron(II) complex
Source: Beilstein J Org Chem. 2026 Sep 23;22:1295–302. doi: 10.3762/bjoc.22.104 (PMC13618351; doi:10.3762/bjoc.22.104)
Supplement: Supplementary file 1 [file Beilstein_J_Org_Chem-22-1295-s001.pdf]

BEILSTEIN JOURNAL OF ORGANIC CHEMISTRY

Supporting Information

for

CO_2 silylation at a tucked-in iron(II) complex

Logan J. Taylor and Marcus W. Drover

Beilstein J. Org. Chem. **2026,** *22,* 1295–1302. doi:10.3762/bjoc.22.104

Copies of spectra and crystallography details

License and Terms: This is a supporting information file under the terms of the Creative Commons Attribution License (https://creativecommons.org/licenses/by/4.0). Please note that the reuse, redistribution and reproduction in particular requires that the author(s) and source are credited and that individual graphics may be subject to special legal provisions.
The license is subject to the *Beilstein Journal of Organic Chemistry* terms and conditions: (https://www.beilstein-journals.org/bjoc/terms)

Table of contents

1. Spectroscopic data S2
2. Mass spectrometry S18
3. Crystallographic details S20
4. References S22

1. Spectroscopic data:

Figure S1: 2, ^{1}H NMR, C_6D_6, 400 MHz, 298 K.

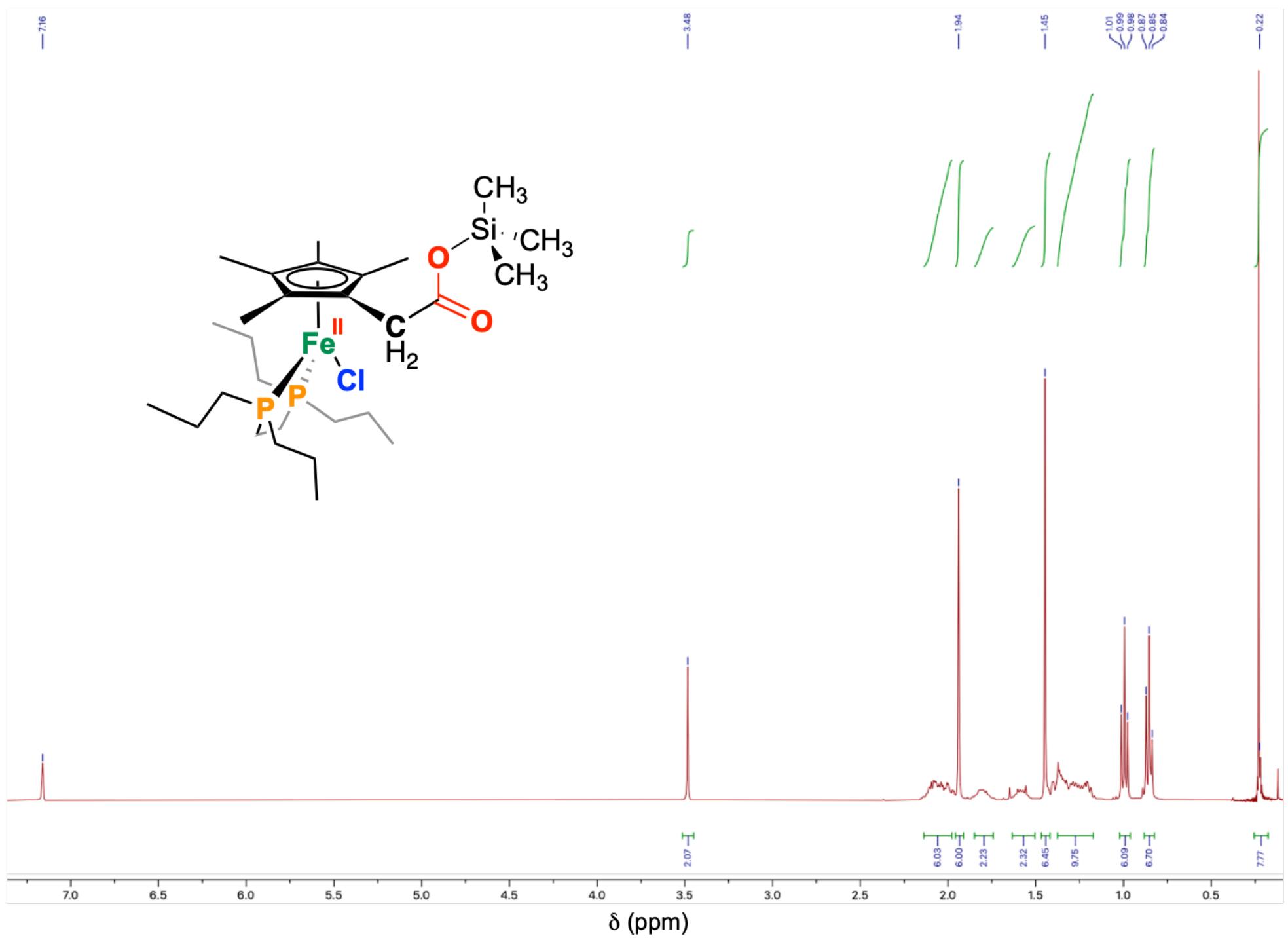

Figure S2: 2, $^{31}P\{^{1}H\}$ NMR, C_6D_6, 162 MHz, 298 K.

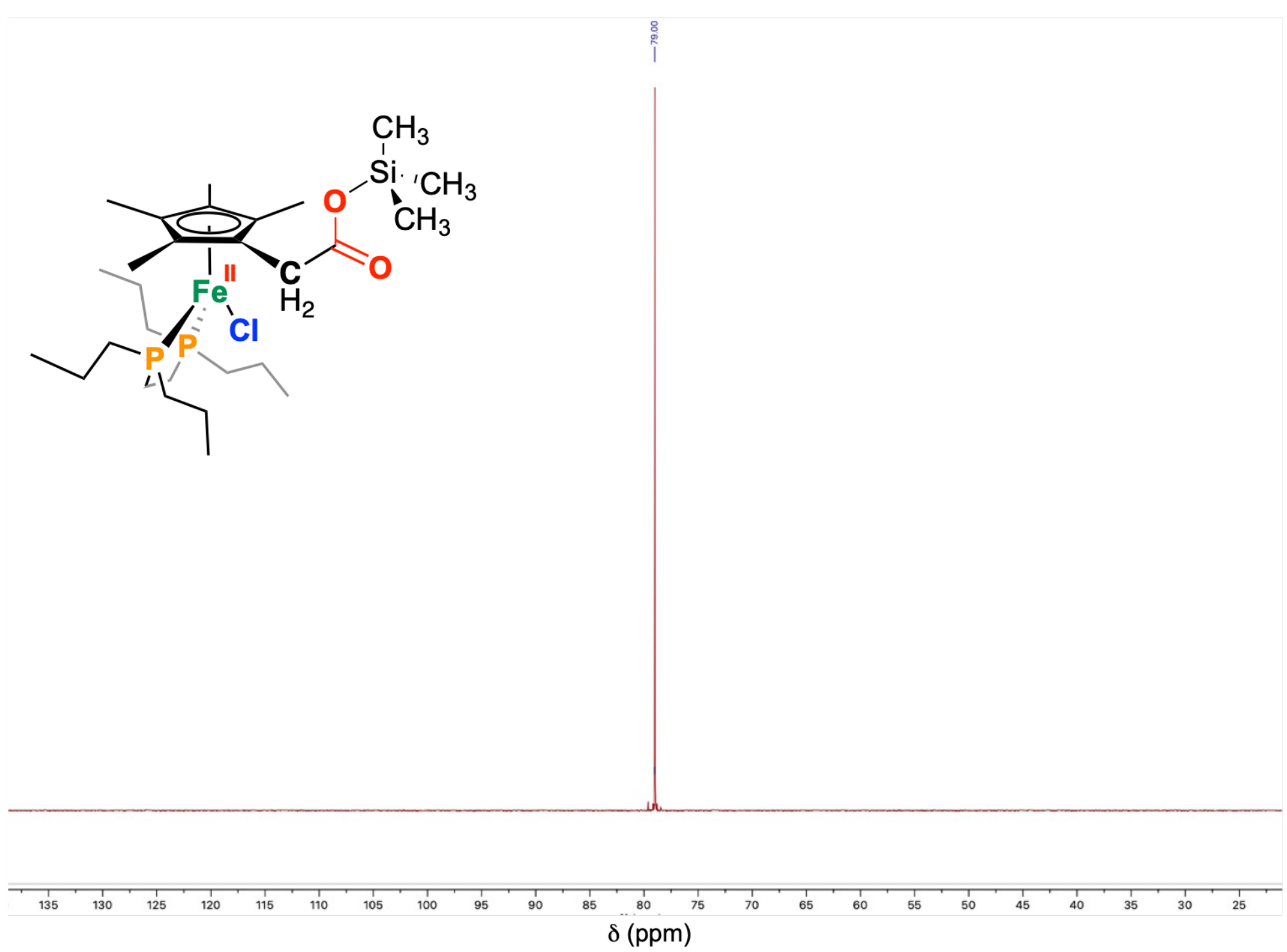

Figure S3: 2, $^{1}H,^{29}Si$ HMBC NMR, C_6D_6, 400 MHz, 298 K.

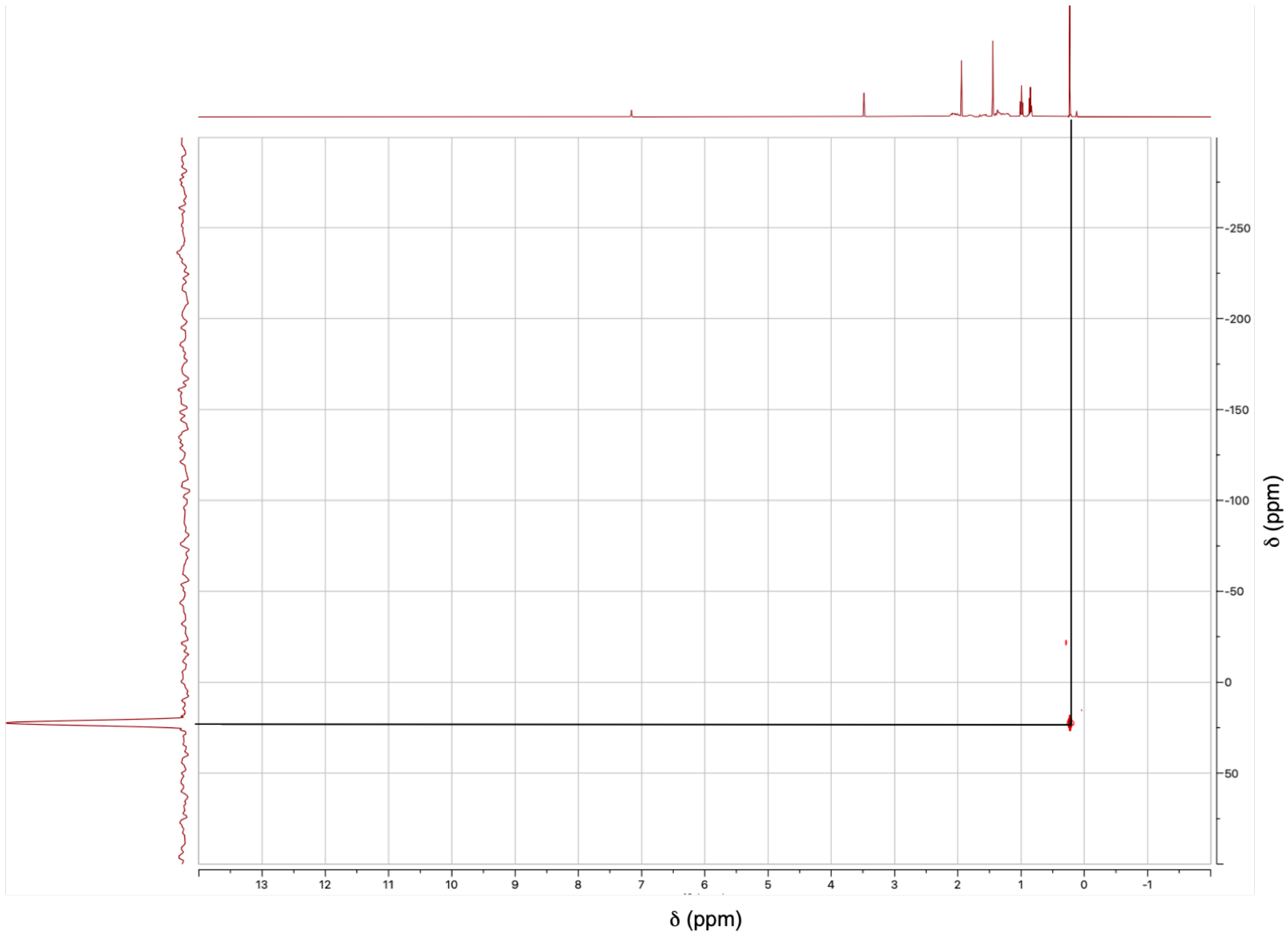

Figure S4: 2, $^{13}C\{^{1}H\}$ NMR, C_6D_6, 101 MHz, 298 K.

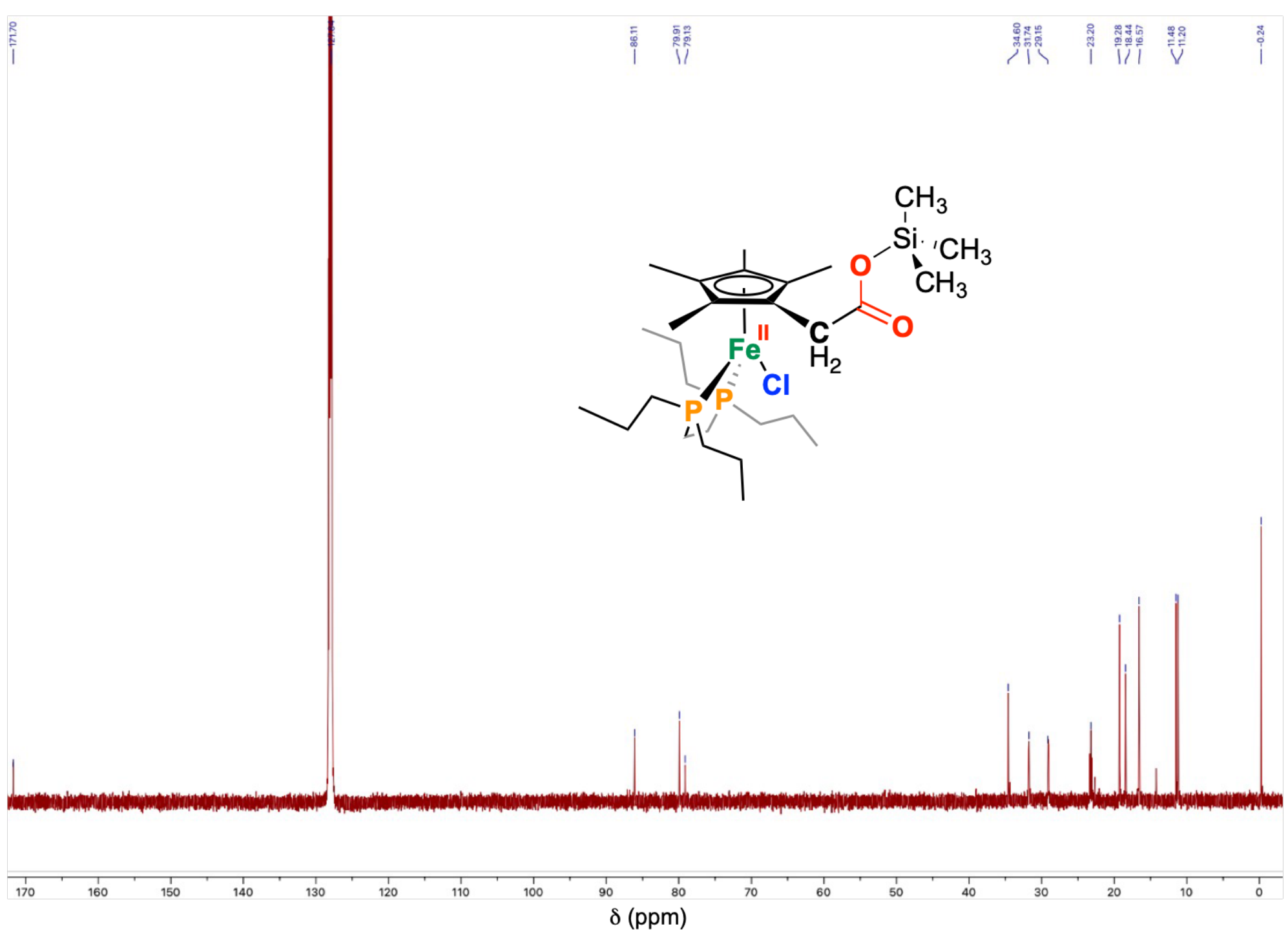

Figure S5: 2, FTIR (benzene film). υ = 1716 cm^{-1} (C=O).

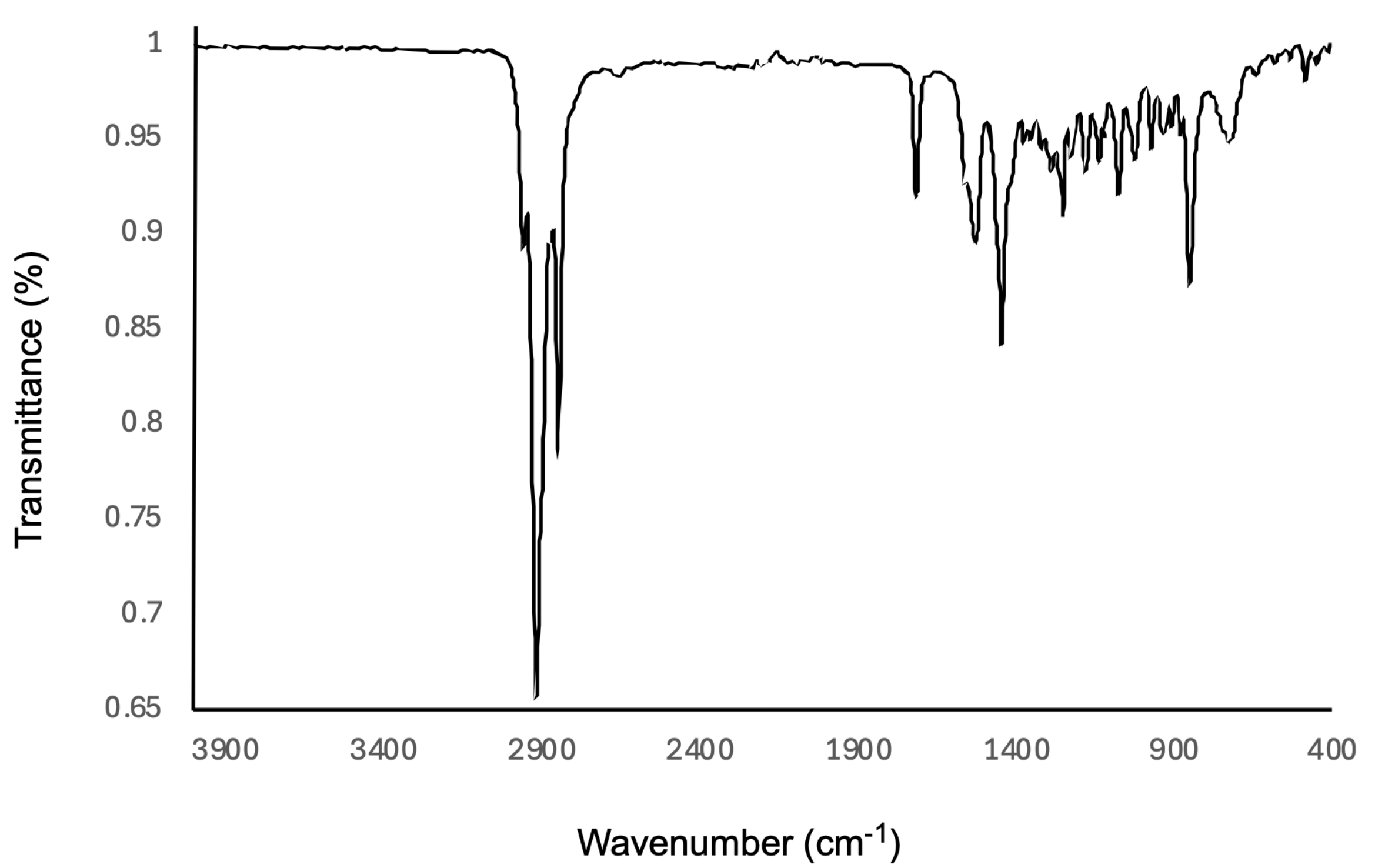

Figure S6: 3, $^{31}P\{^{1}H\}$ NMR, C_6D_6, 162 MHz, 298 K.

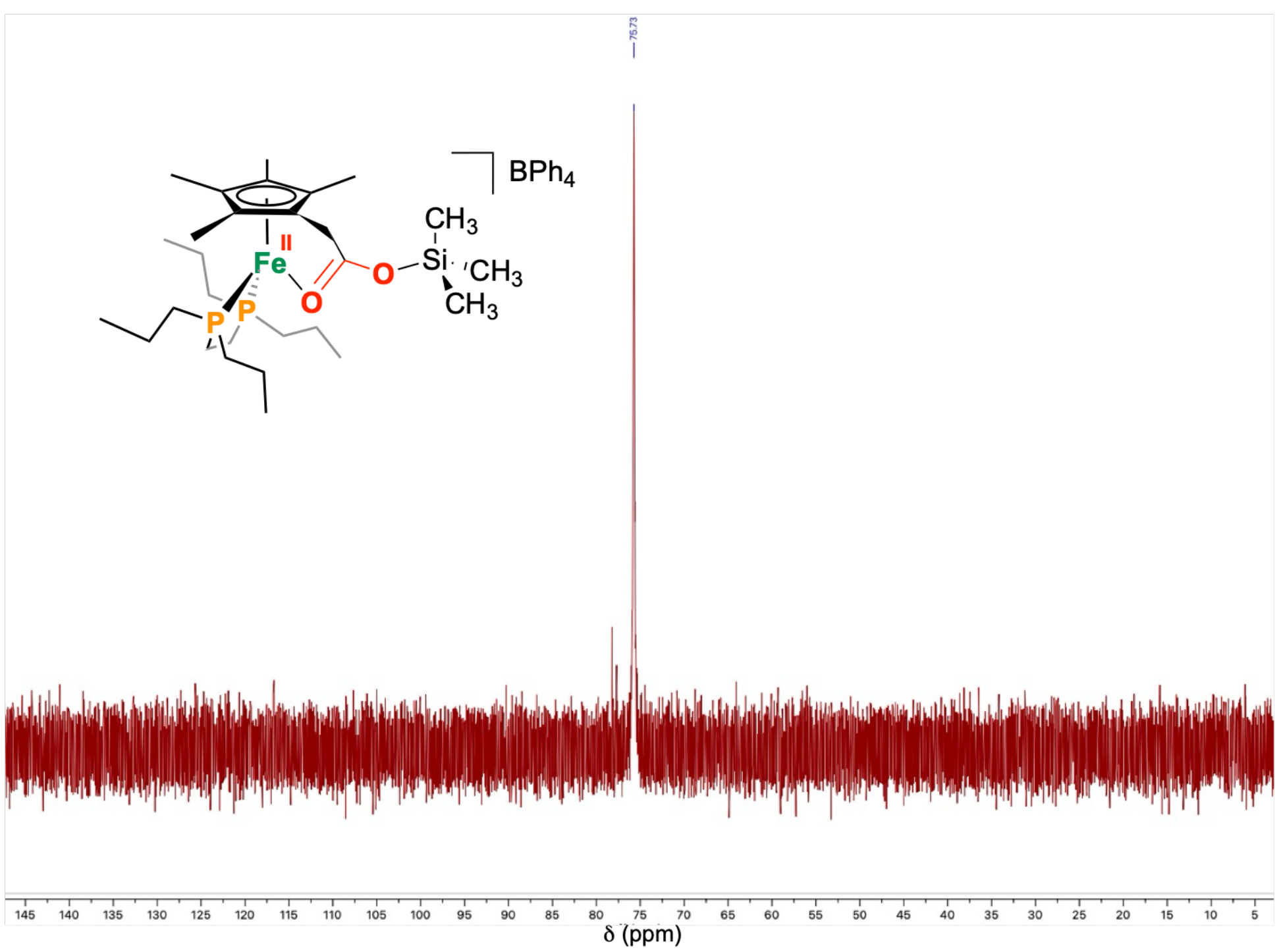

Figure S7: 3, $^{31}P\{^1H\}$ VT NMR, THF−H_8, 243 MHz, 298 K. (25, 0, −25, −50, −75 °C)

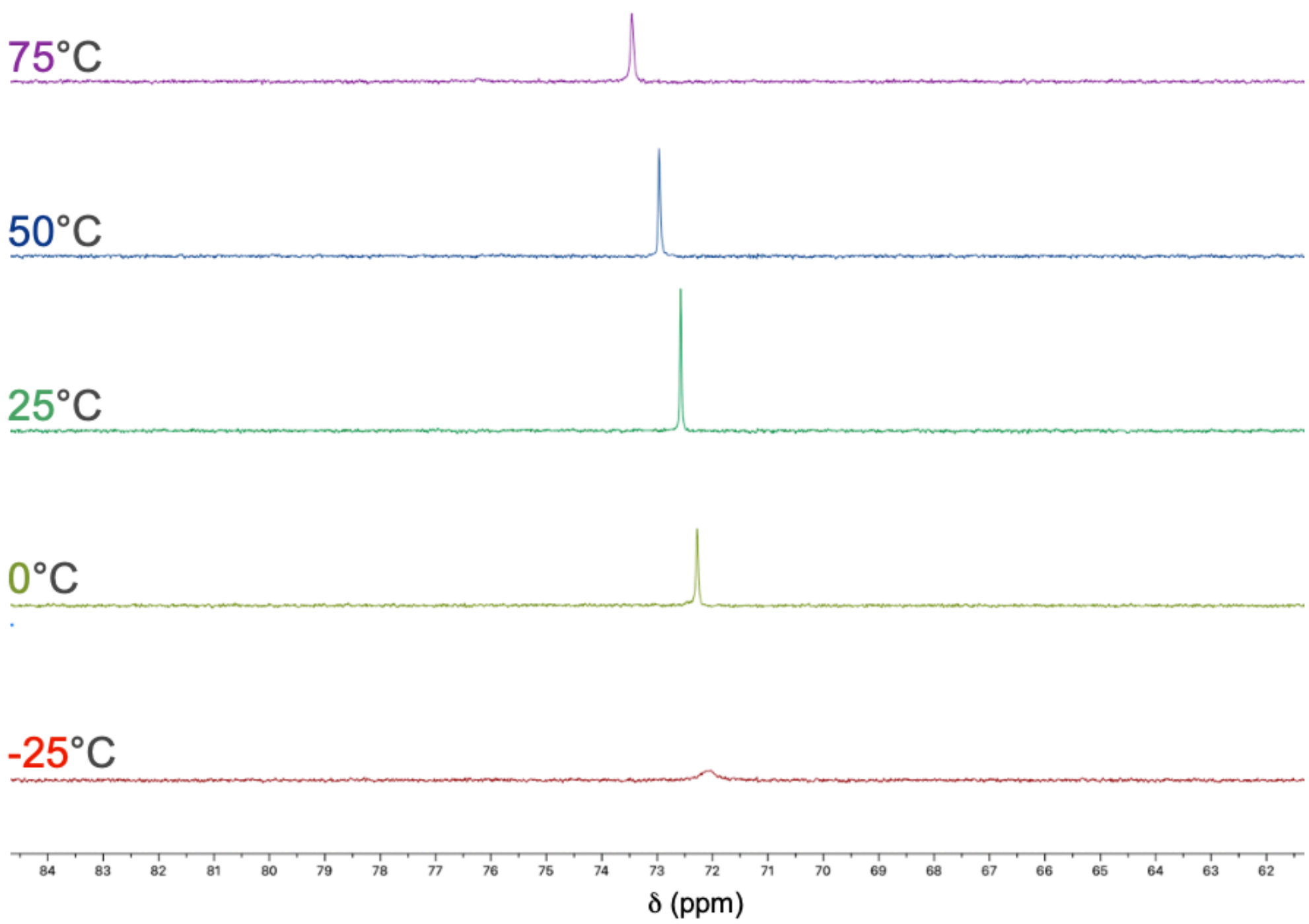

Figure S8: 3, FTIR (ATR, THF Film) shows no υ(N_2) stretch.

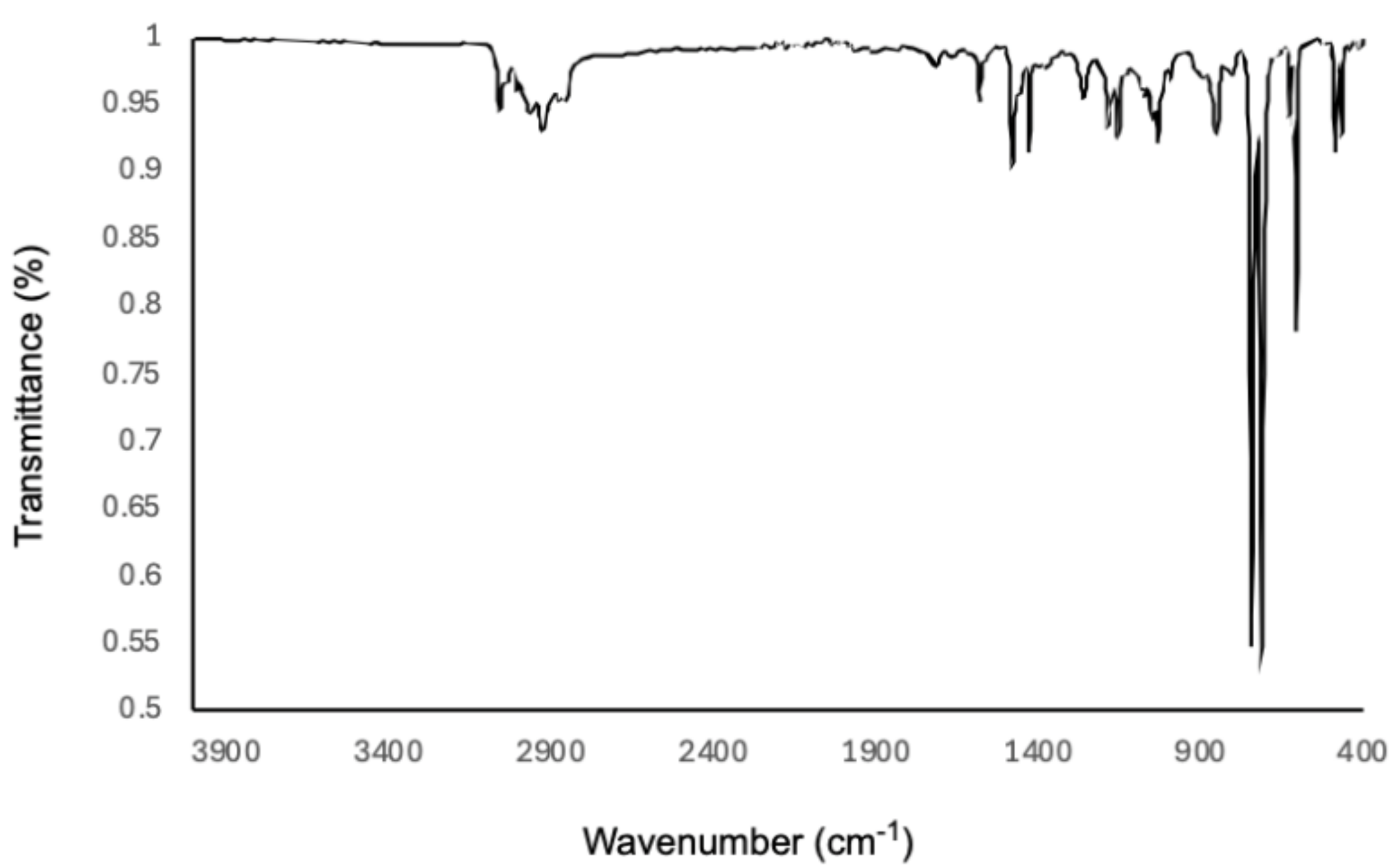

Figure S9: 4, ^{1}H NMR, THF−d_8, 400 MHz, 298 K at t = 5 min. (*=unknown impurity)

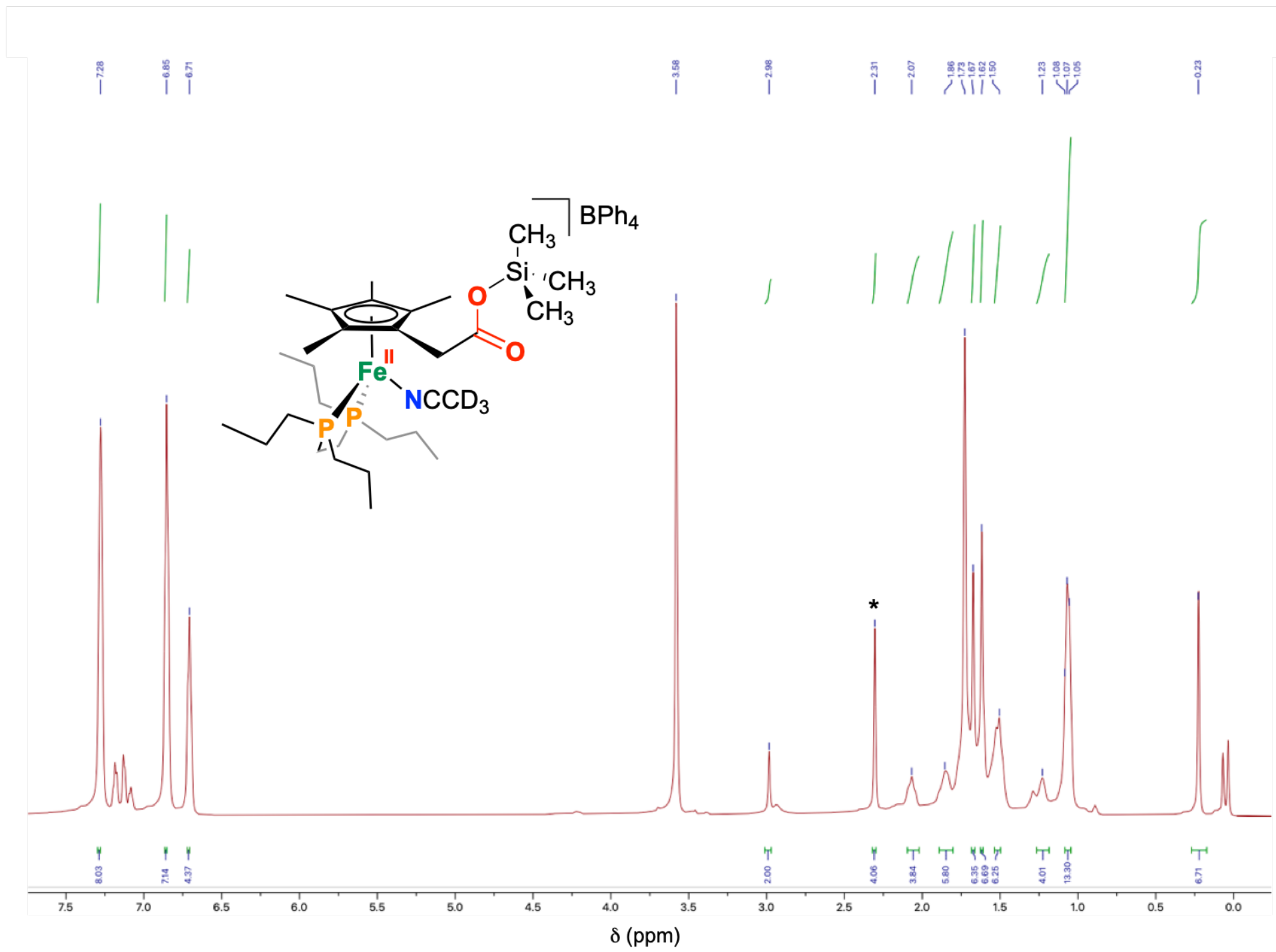

Figure S10: 4, $^{31}P\{^{1}H\}$ NMR, THF−d_8, 162 MHz, 298 K at t = 5 min.

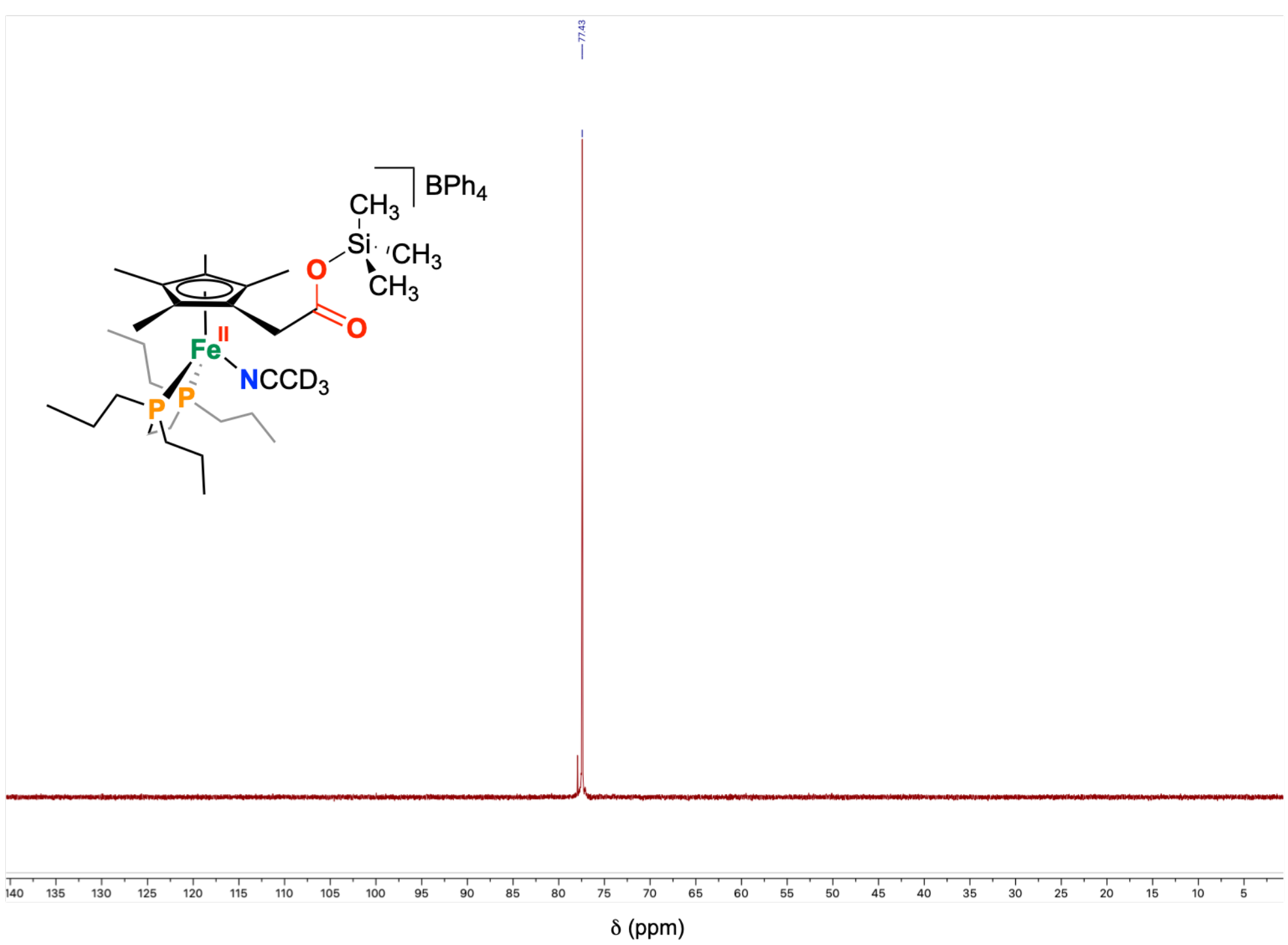

Figure S11: 4, ^{11}B NMR, THF−d_8, 128 MHz, 298 K at t = 5 min.

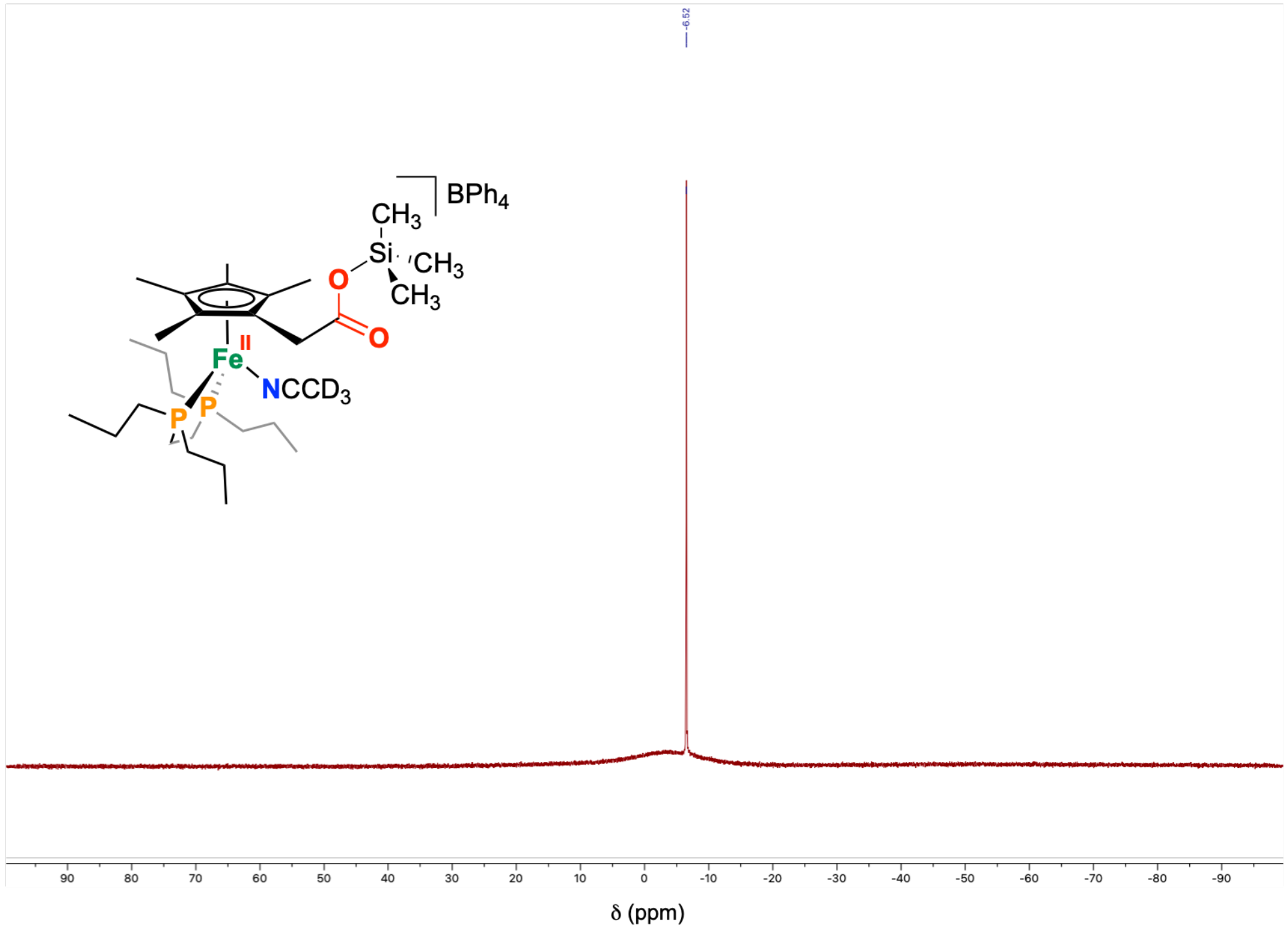

Figure S12: 4, ^{1}H NMR, THF−d_8, 400 MHz, 298 K at t = 12 h showing decomposition to **5**.

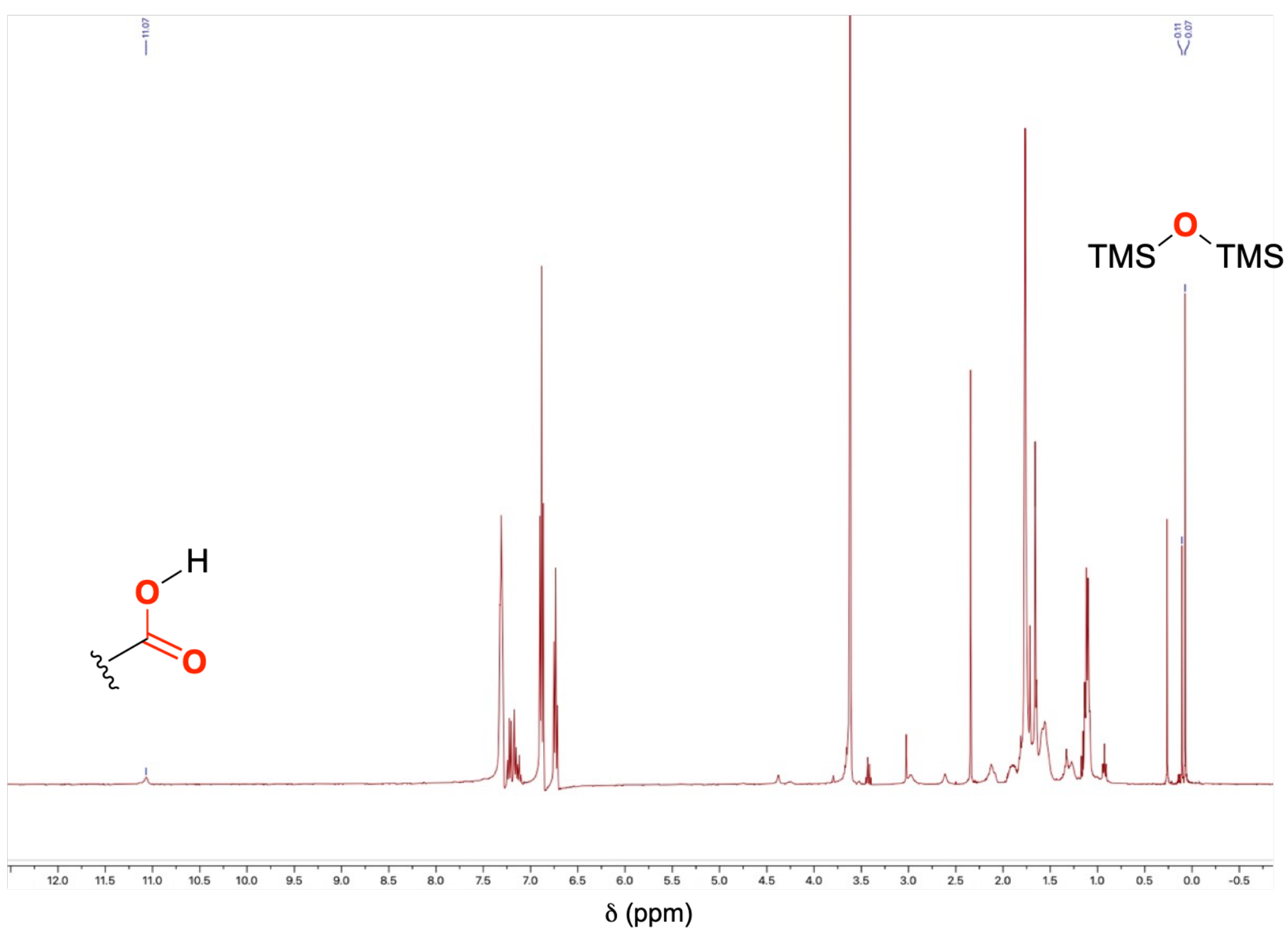

Figure S13: 4, $^{31}P\{^1H\}$ NMR, THF−d_8, 162 MHz, 298 K at t = 12 h showing decomposition to **5**.

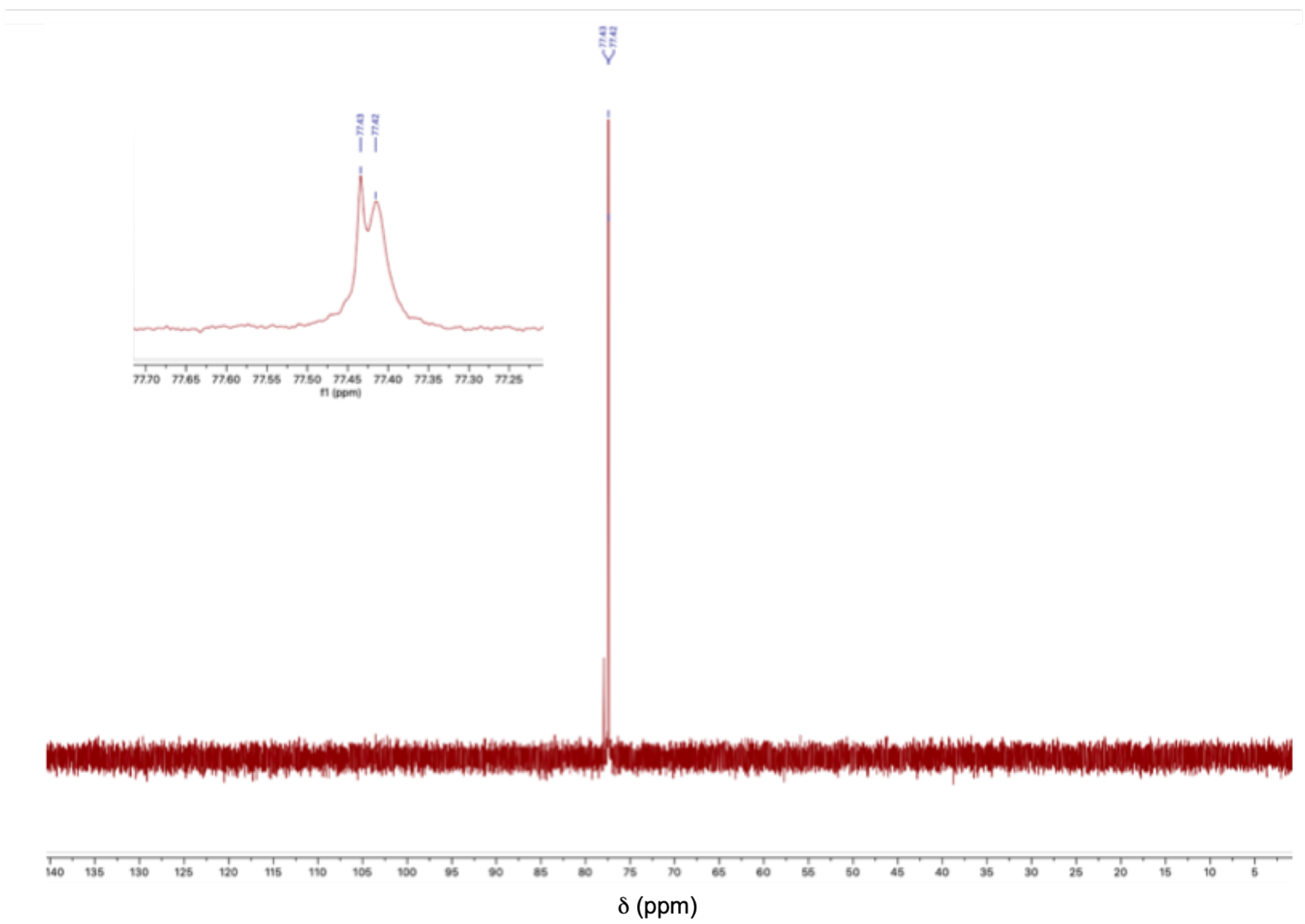

Figure S14: 4, ^1H NMR, THF−d_8, 400 MHz, 298 K at t = 5 min (bottom) 12 h (top).

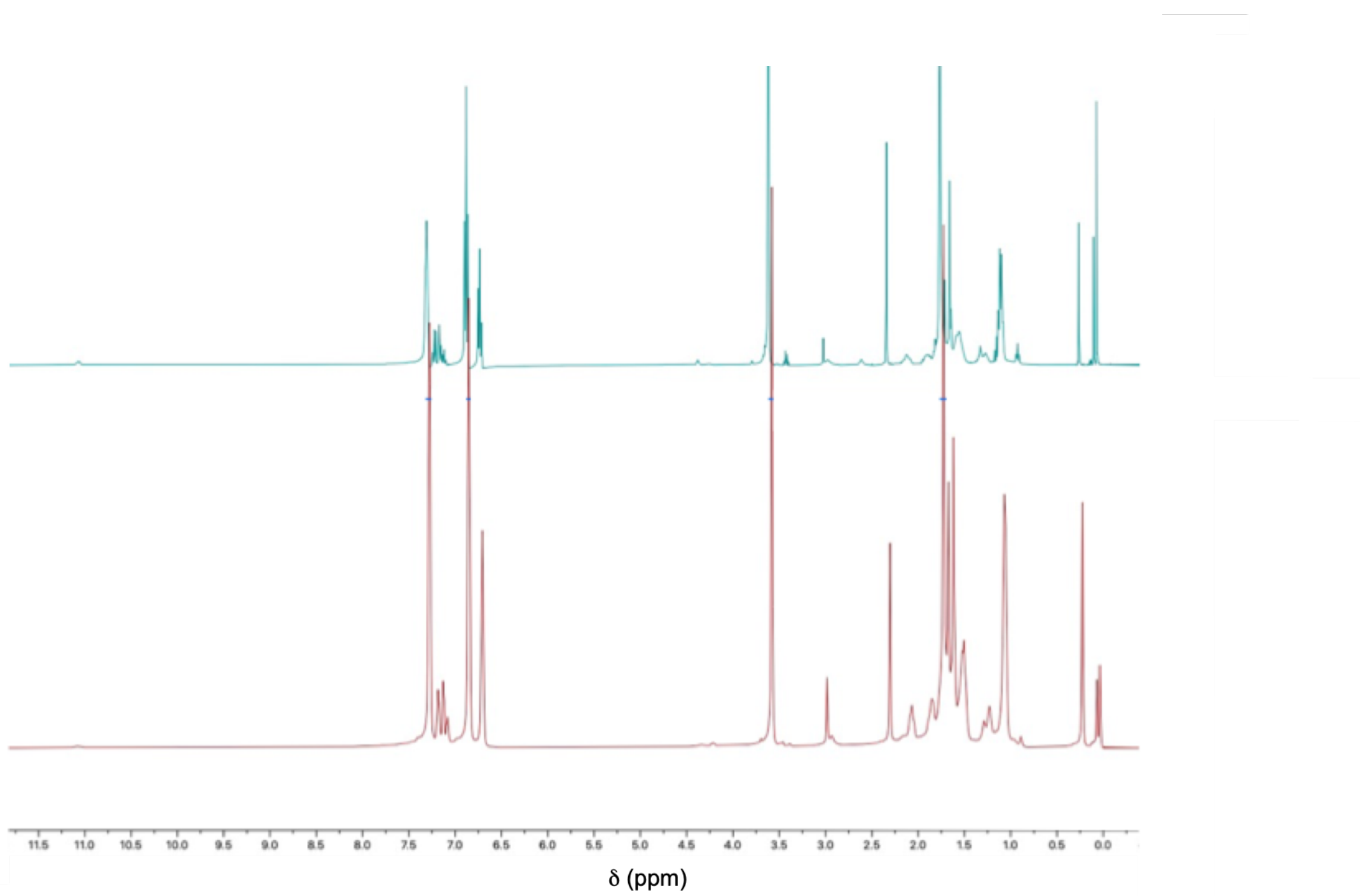

Figure S15: 4, $^{31}P\{^{1}H\}$ NMR, THF−d_8, 162 MHz, 298 K at t = 5 min (bottom) 12 h (top).

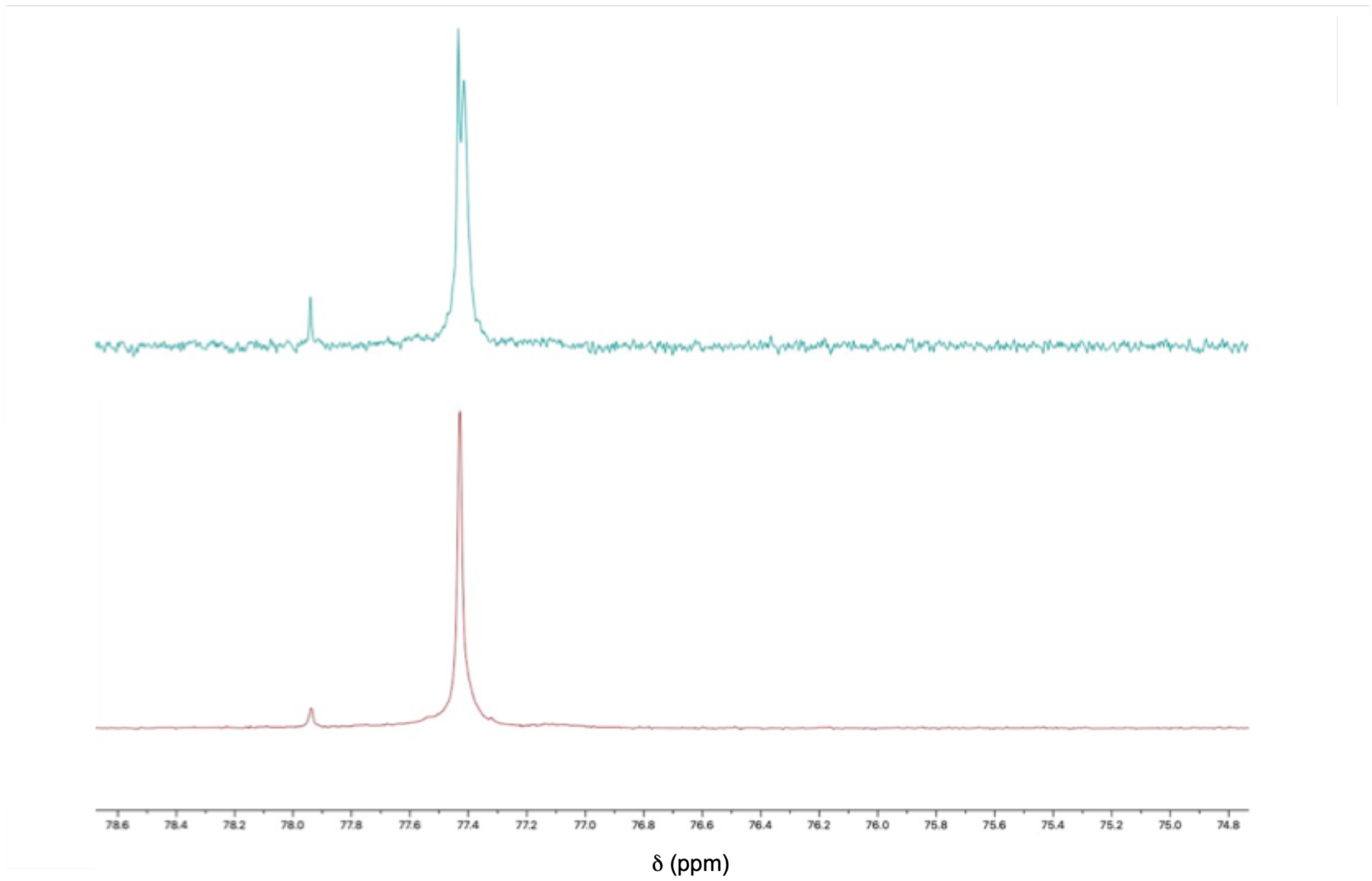

Figure S16: 4, FTIR (ATR, Acetonitrile film): υ = 2243 cm^{-1} (C≡N).

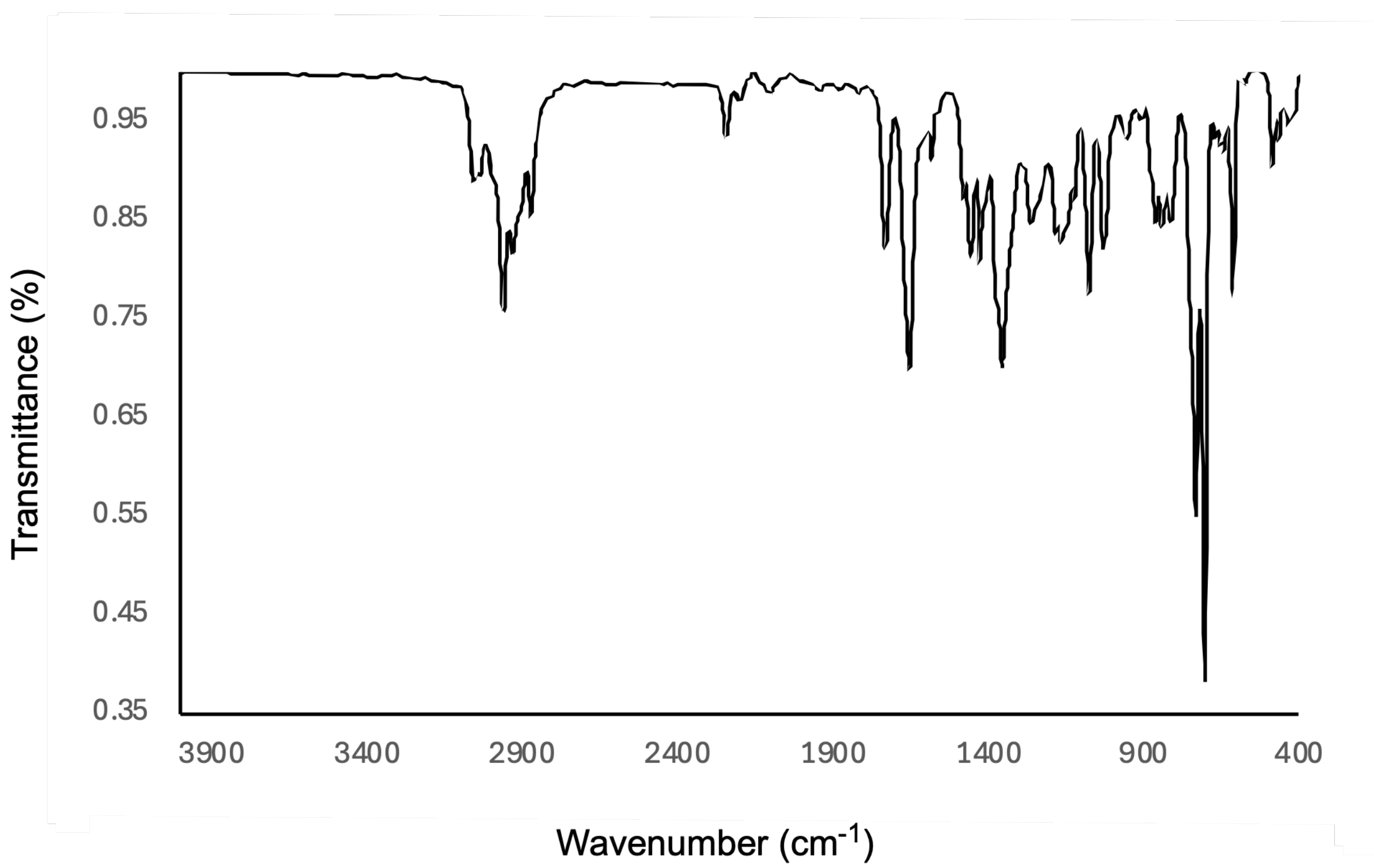

Figure S17: Reaction of **2** with 5 equiv. $HBCy_2$, ^1H NMR, C_6D_6, 400 MHz, 298 K.

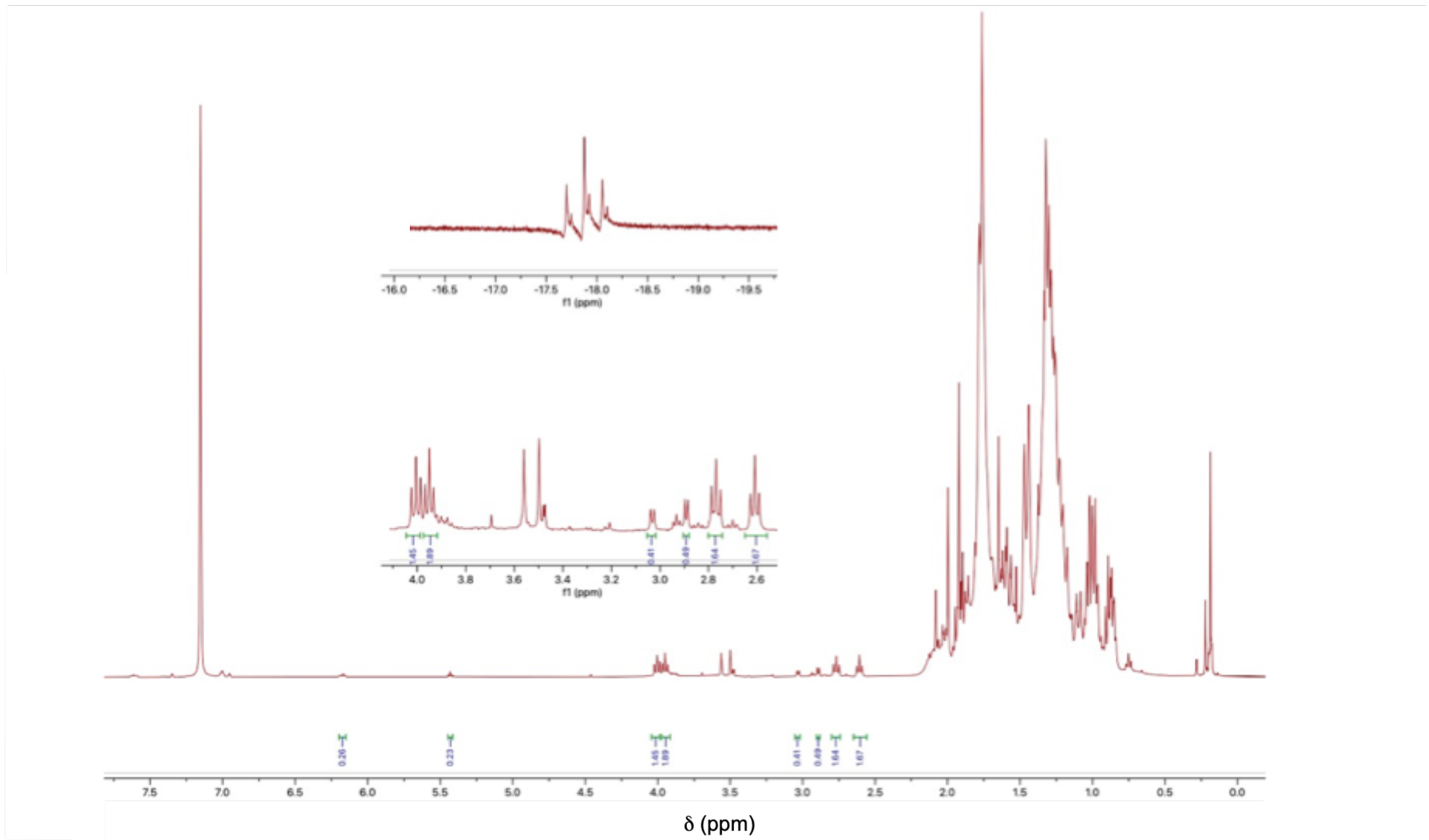

Figure S18: ^1H 1D TOCSY NMR from Figure S17, C_6D_6, 600 MHz, 298 K (Irradiation at δ_H = 3.95).

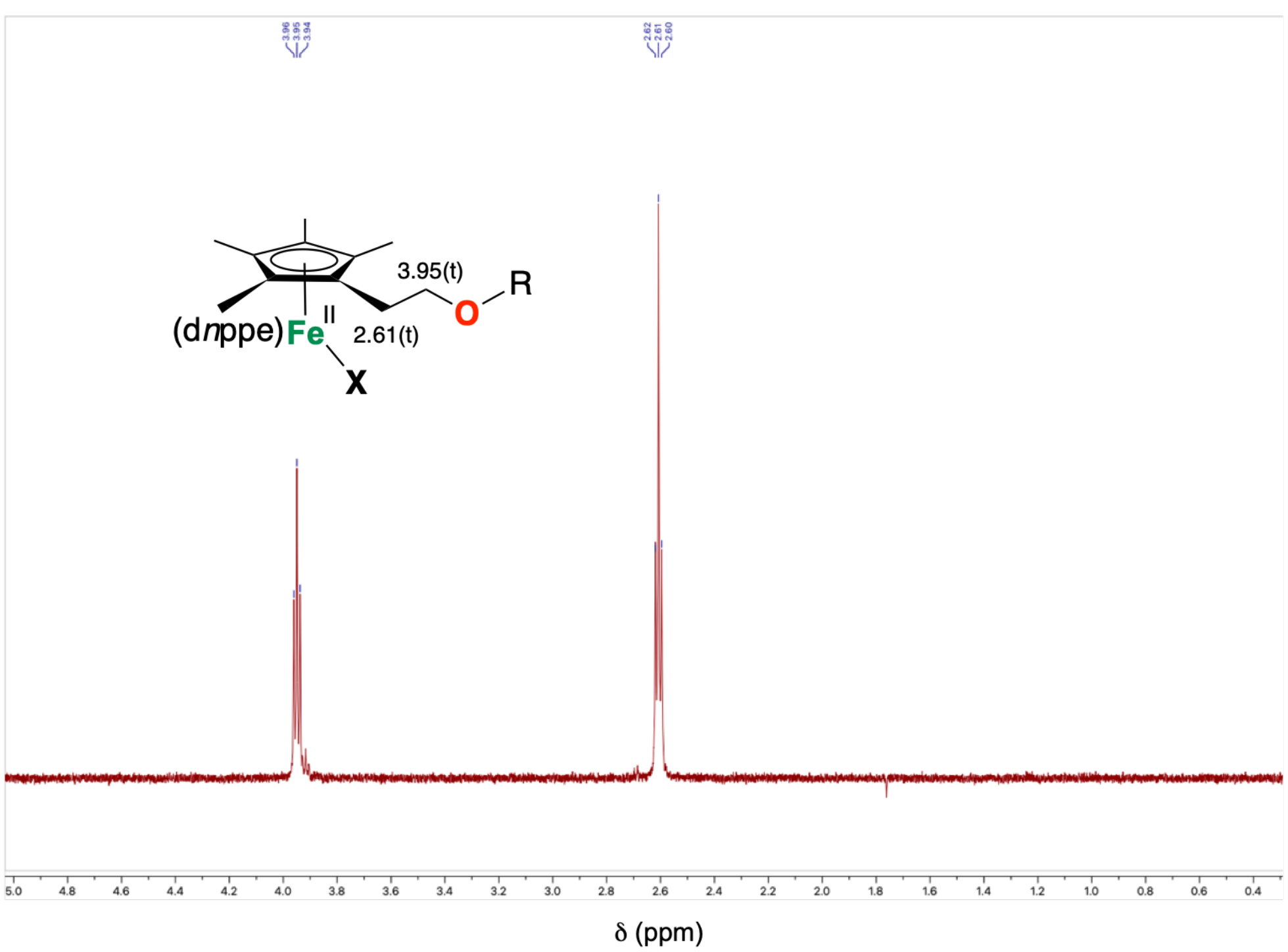

Figure S19: ^{1}H 1D TOCSY NMR from Figure S17, C_6D_6, 600 MHz, 298 K (Irradiation at δ_H = 5.43).

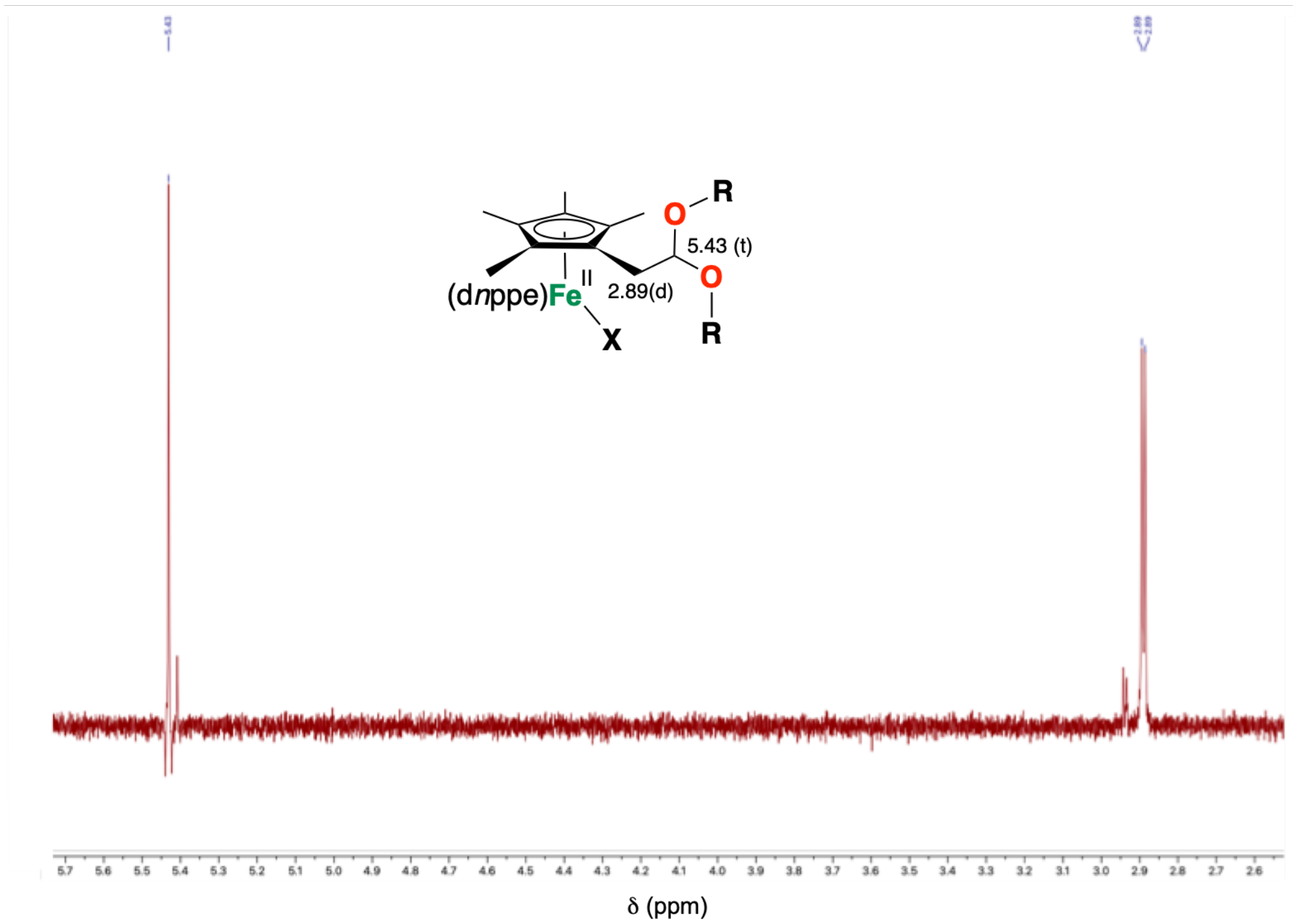

Figure S20: ^{1}H 1D TOCSY NMR from Figure S17, C_6D_6, 600 MHz, 298 K (Irradiation at δ_H = 6.17 ppm).

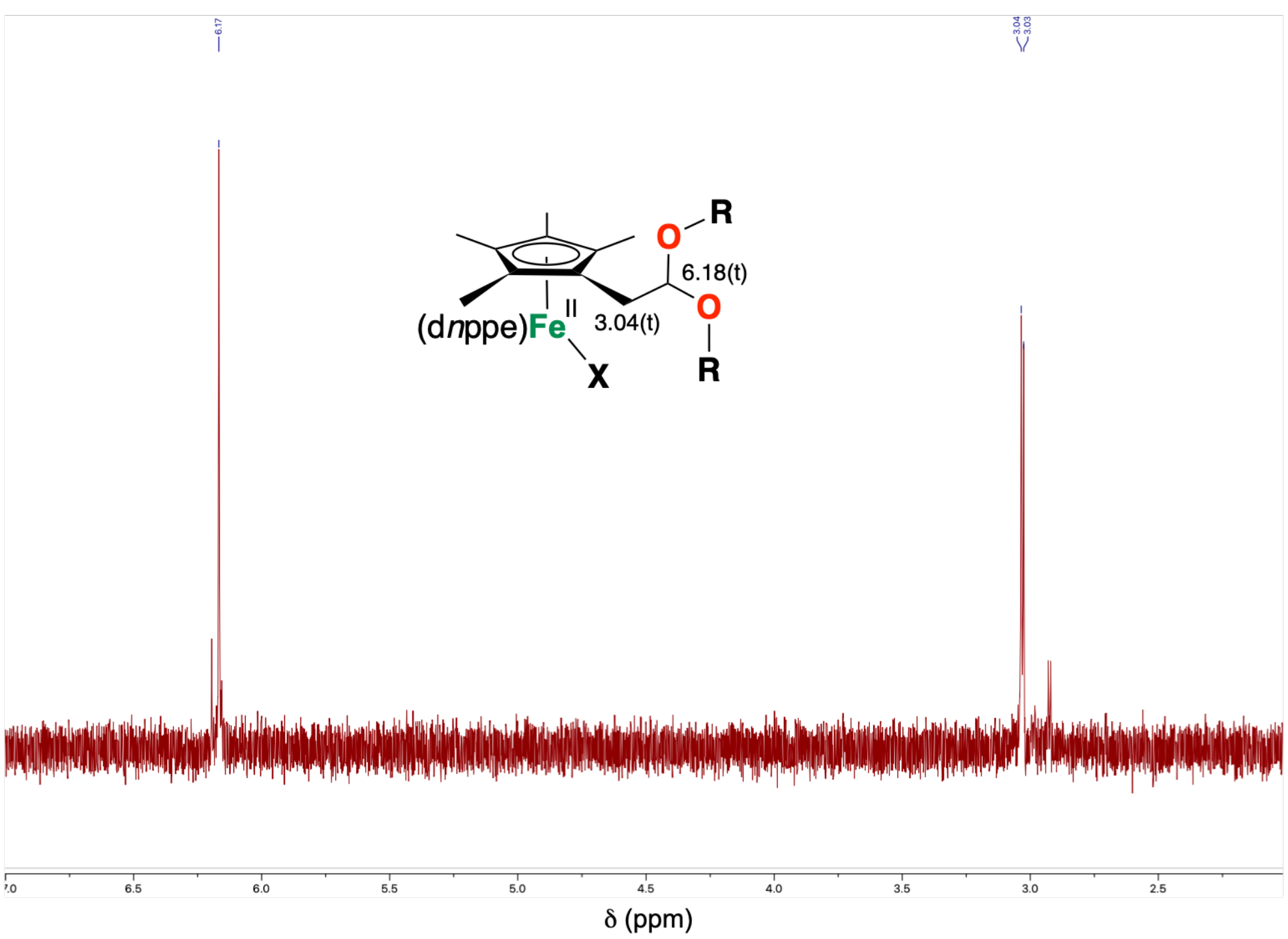

Figure S21: Reaction of **2** with 5 equiv. $HBCy_2$, $^{31}P\{^1H\}$ NMR, C_6D_6, 162 MHz, 298 K.

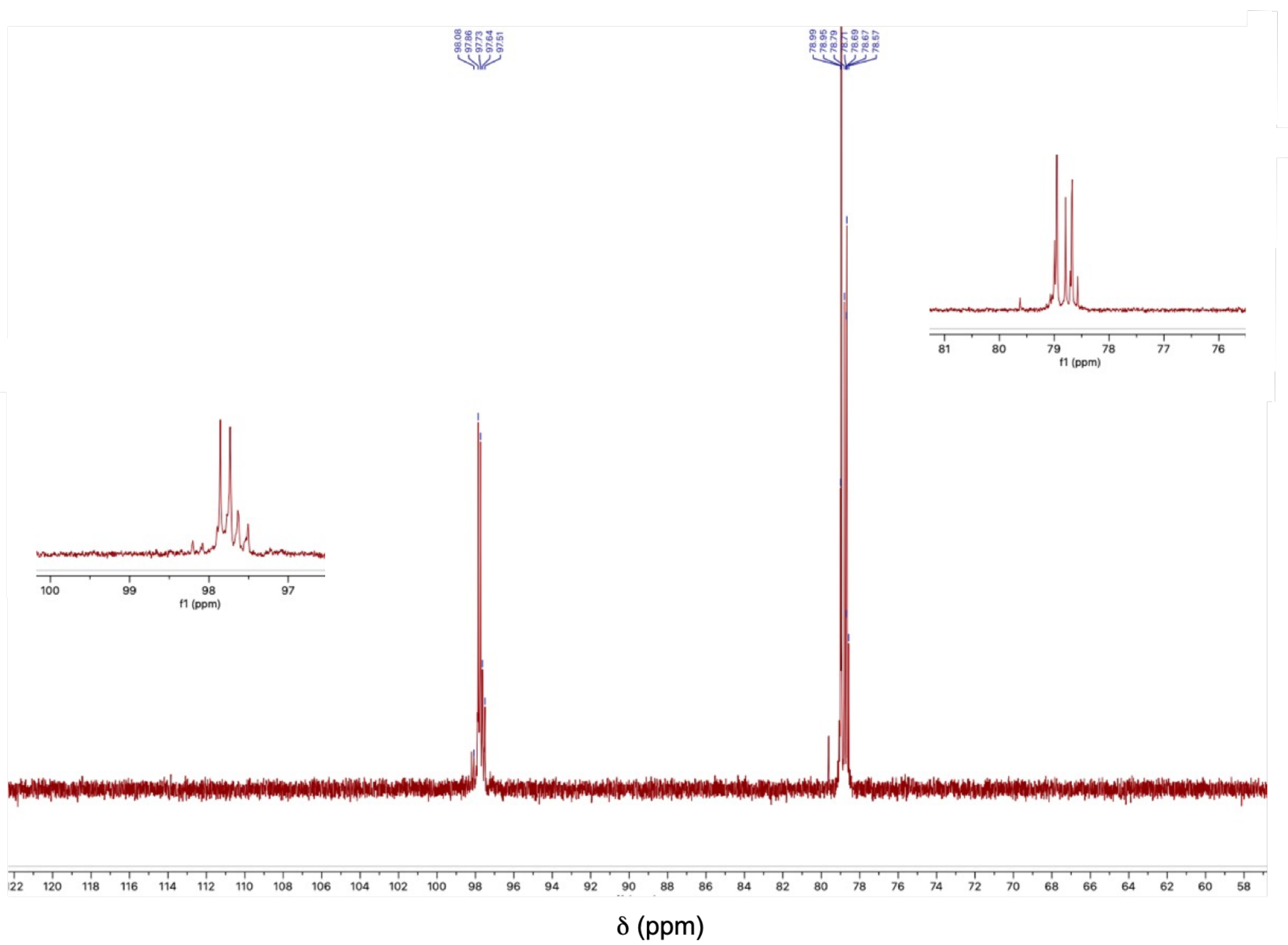

Figure S22: Reaction of **2** with 5 equiv. $HBCy_2$, ^{11}B NMR, C_6D_6, 128 MHz, 298 K. δ_B = 45−55 ppm (RO_2B-R).

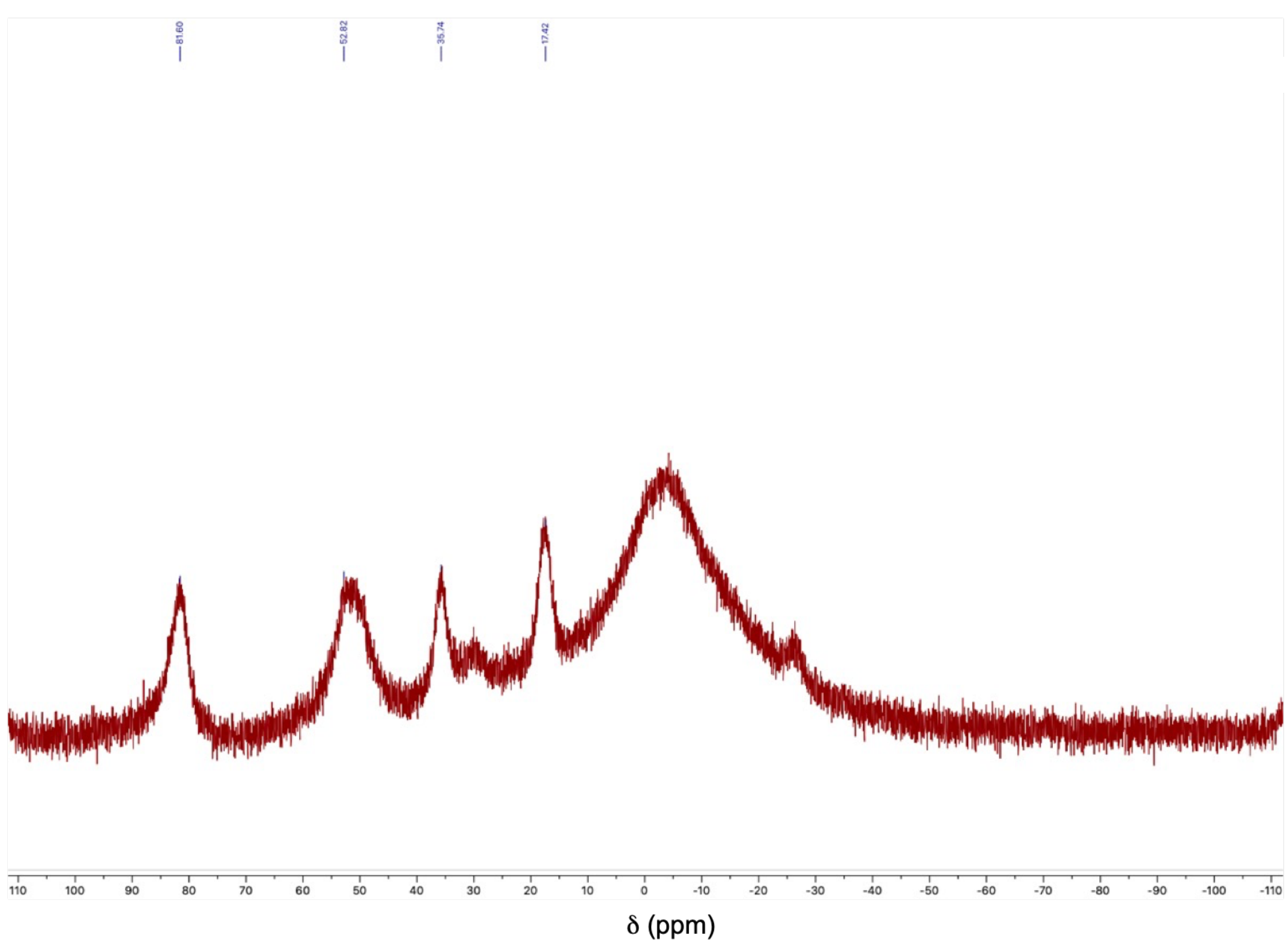

Figure S23: Reaction of **2** with 5 equiv. $HBCy_2$, ^1H, ^1H COSY NMR, C_6D_6, 600 MHz, 298 K showing triplet and acetal cross peaks.

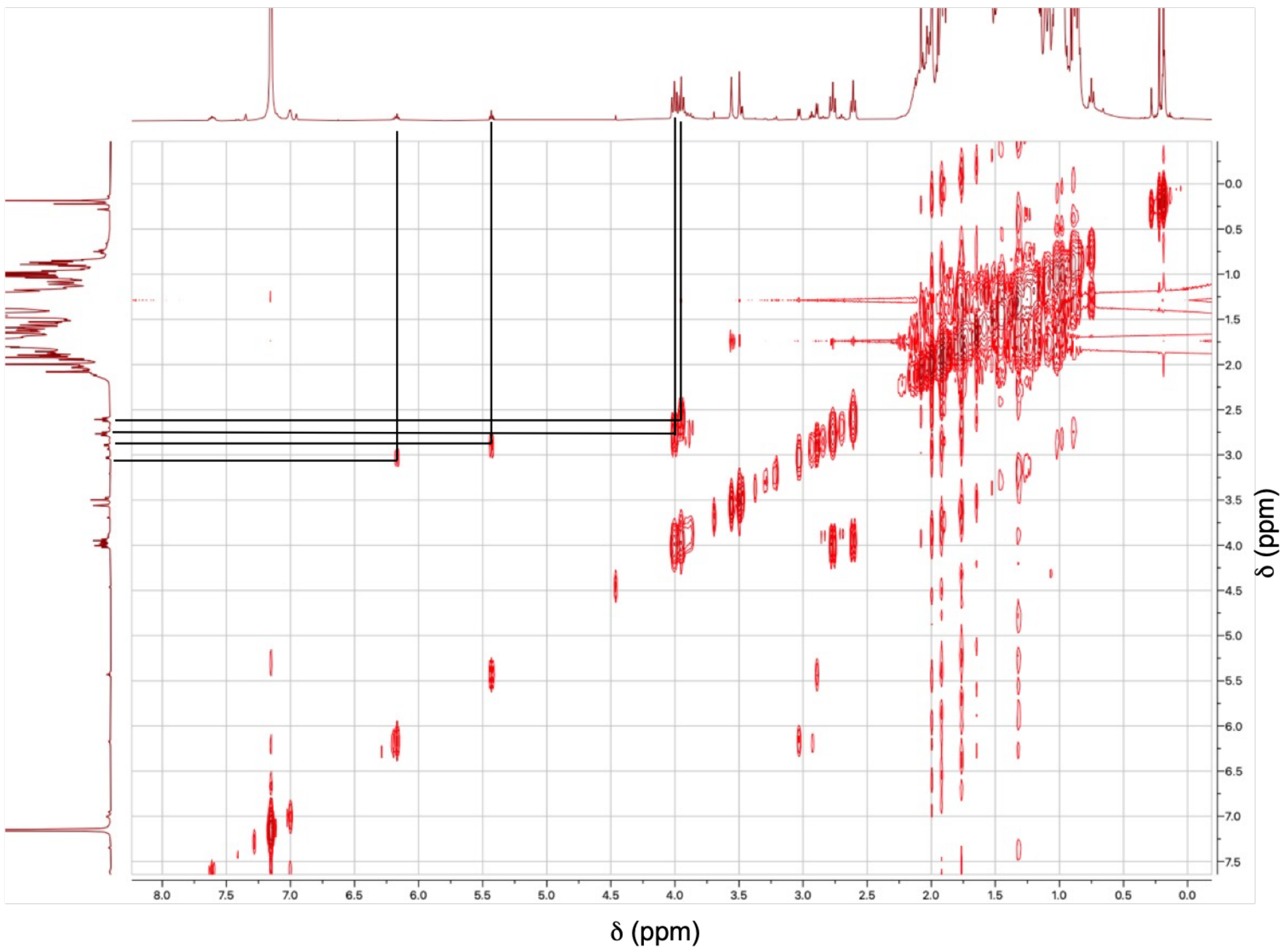

Figure S24: Reaction of **2** with 5 equiv. $HBCy_2$, ^1H,^{13}C HSQC NMR, C_6D_6, 600 MHz, 298 K.

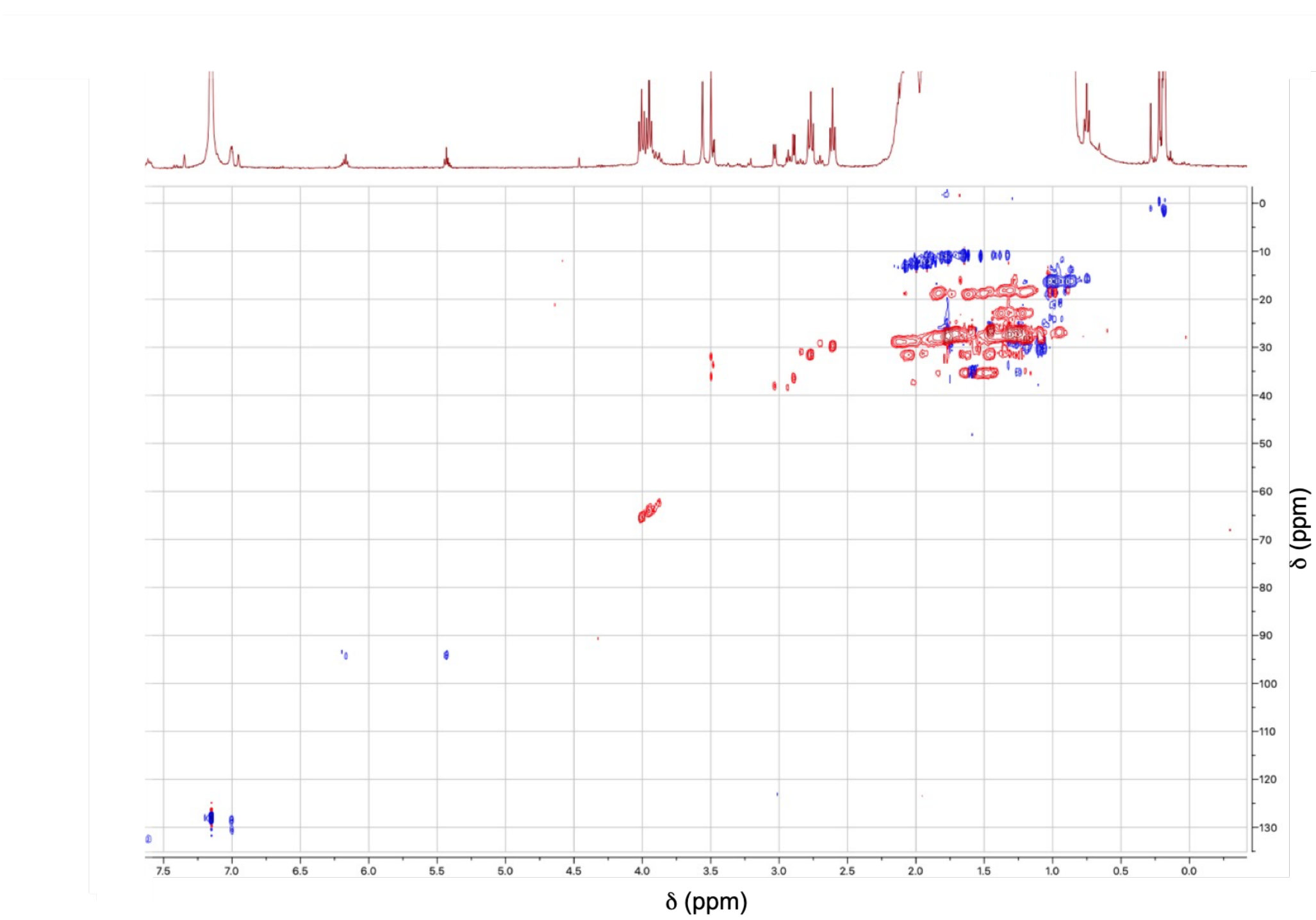

Figure S25: Reaction of **2** with 5 equiv. $HBCy_2$, FT− IR, Benzene Film, 298K. Confirms C=O reduction (absence of a signal 1500−1800 cm^{-1}).

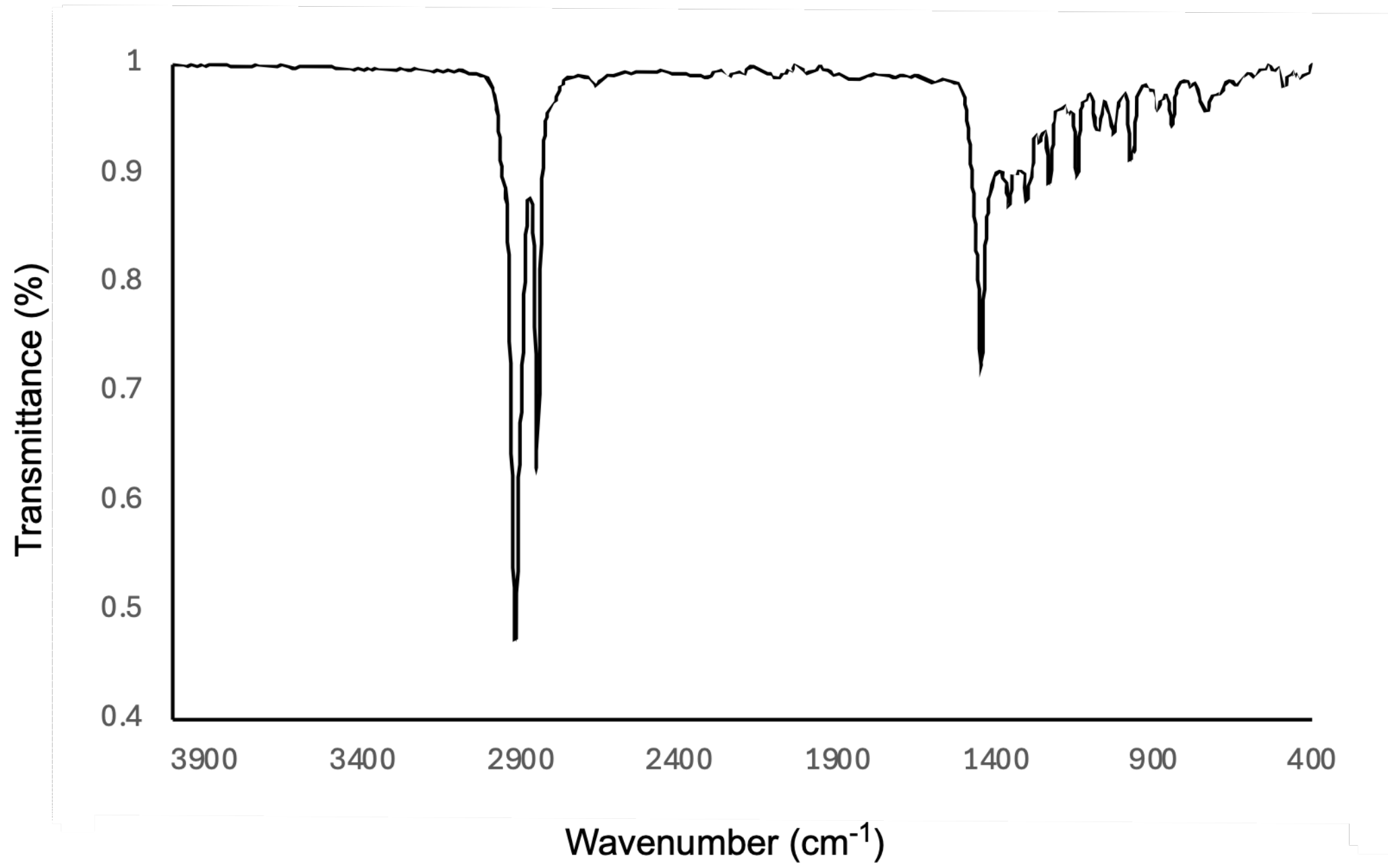

Figure S26: Reaction of **2** with 5 equiv. $HBCy_2$, ^1H NMR, C_6D_6, 400 MHz, 298 K. Reactivity of reduction reaction mixture with $NaBPh_4$ (hexane soluble portion).

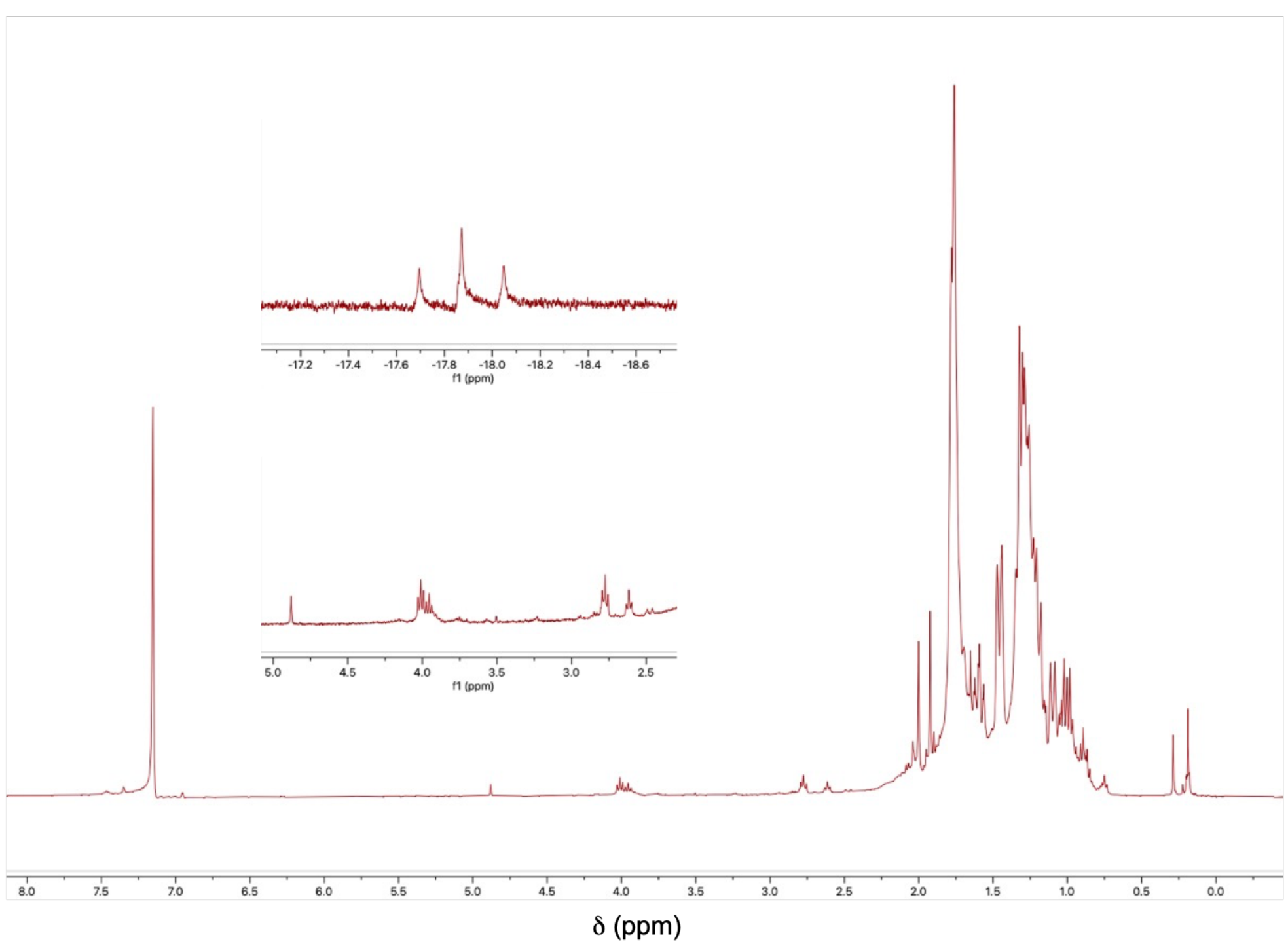

Figure S27: Reaction of **2** with 5 equiv. $HBCy_2$, $^{31}P\{^1H\}$ NMR, C_6D_6, 162 MHz, 298 K. Reactivity of reduction reaction mixture with $NaBPh_4$ (hexane soluble portion).

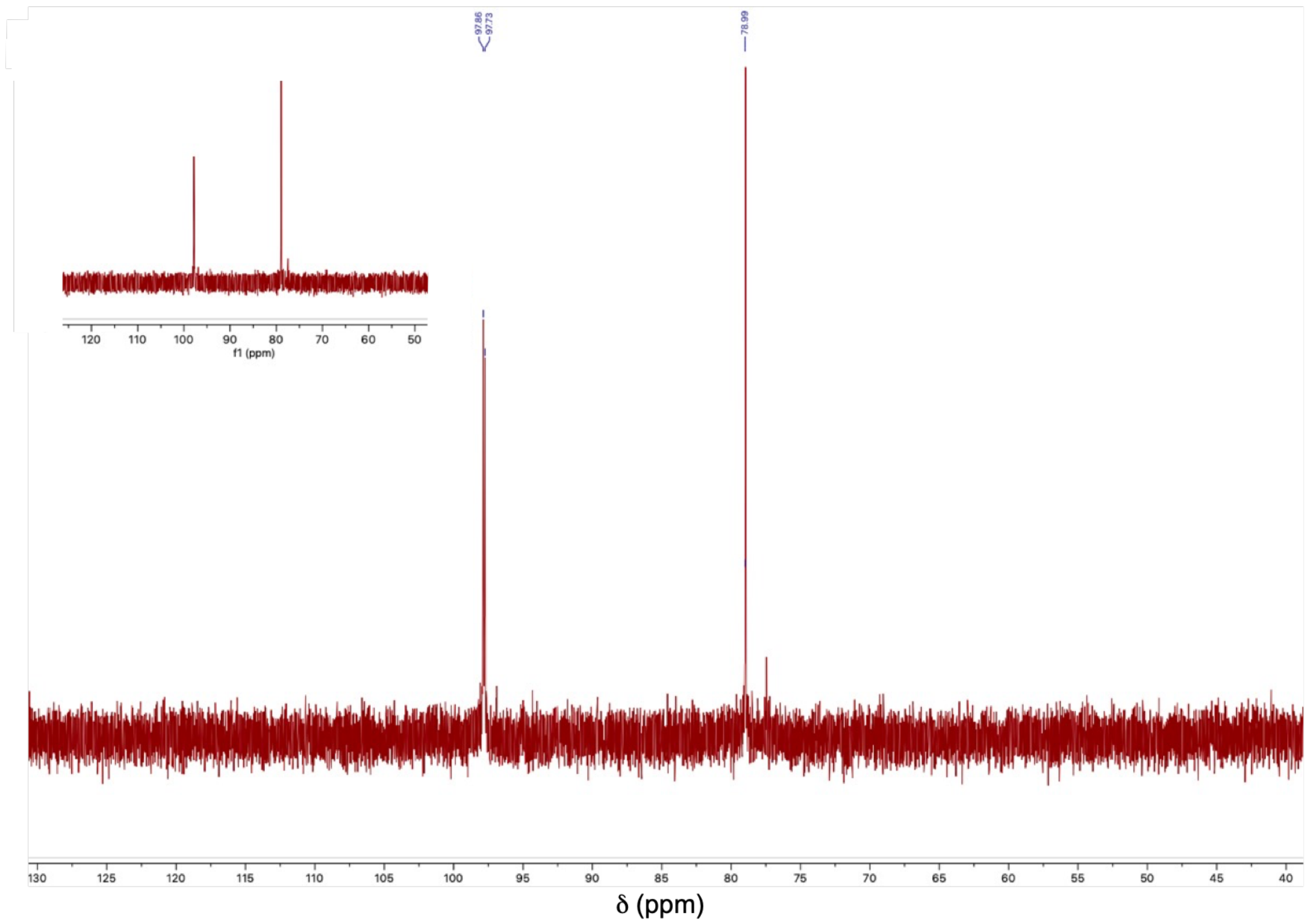

Figure S28: Reaction of **2** with 5 equiv. $HBCy_2$, ^{11}B NMR, C_6D_6, 128 MHz, 298 K. Reactivity of reduction reaction mixture with $NaBPh_4$ (hexane soluble portion).

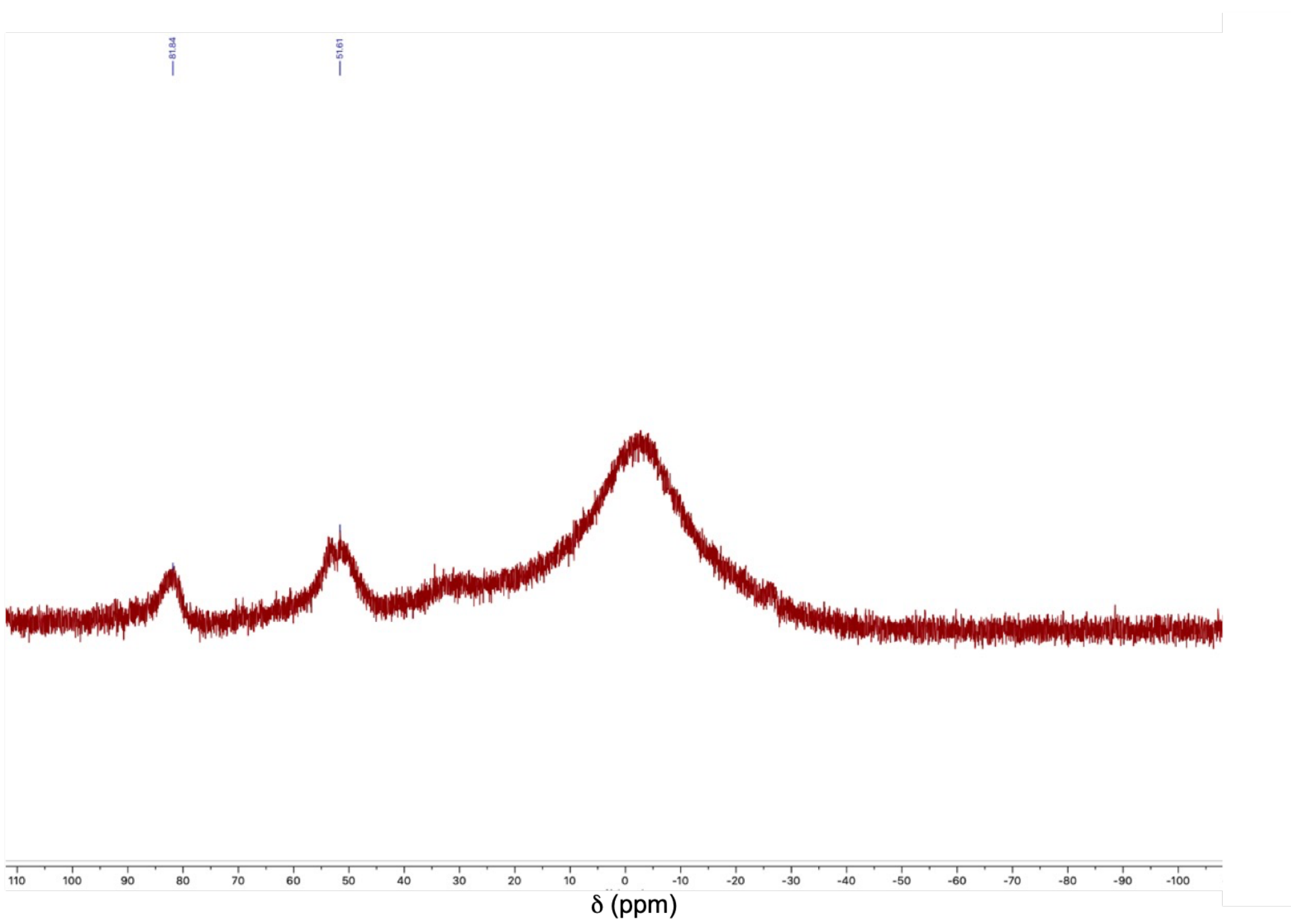

Figure S29: ^{1}H NMR, C_6D_6, 400 MHz, 298K. Reactivity of reduction reaction mixture with $NaBPh_4$ (hexane soluble portion) (bottom) stacked with pre−cleaning (top).

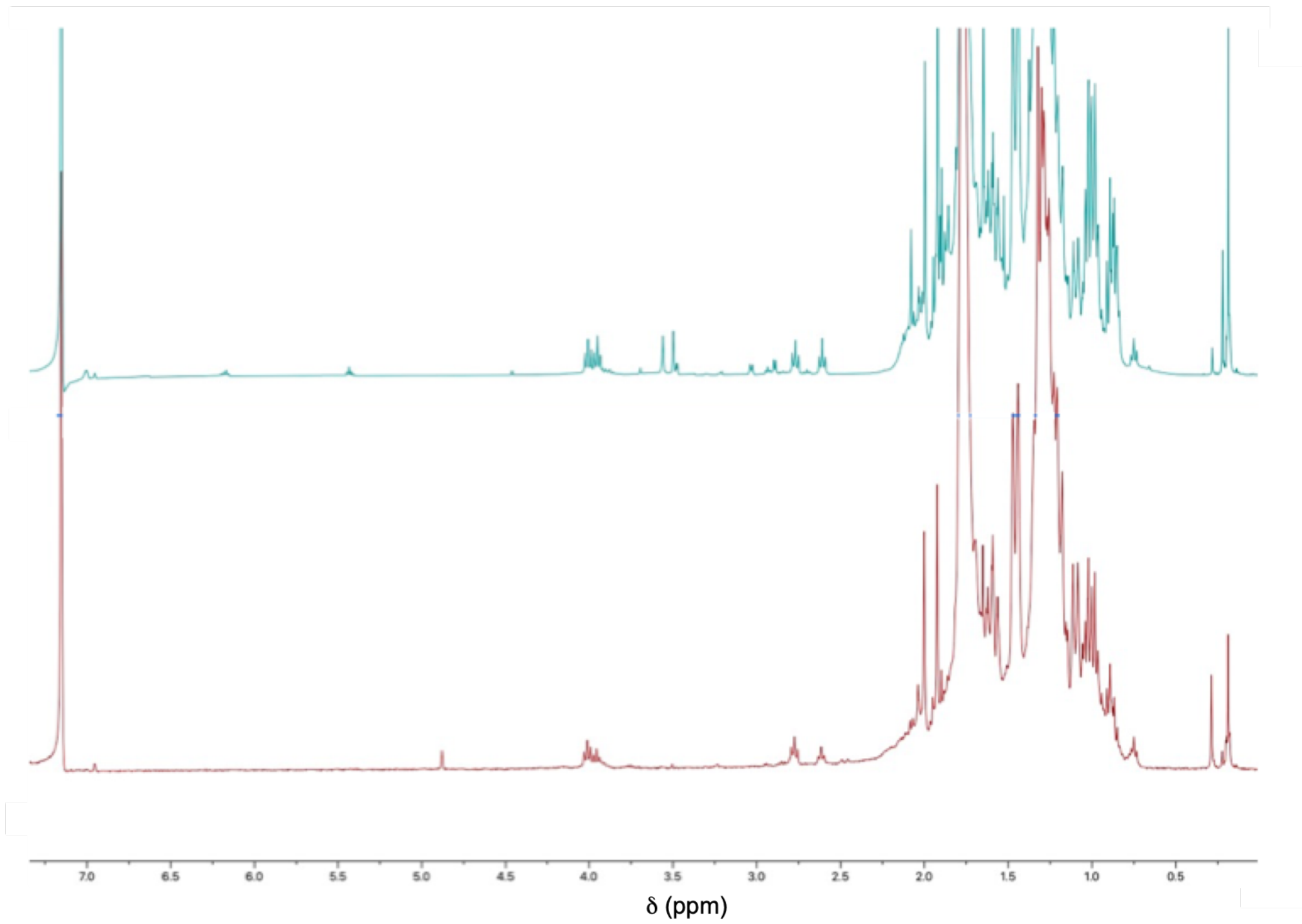

Figure S30: ^{31}P{^{1}H} NMR, C_6D_6, 162 MHz, 298 K. Reactivity of reduction reaction mixture with $NaBPh_4$ (hexane soluble portion) (bottom) stacked with pre−cleaning (top).

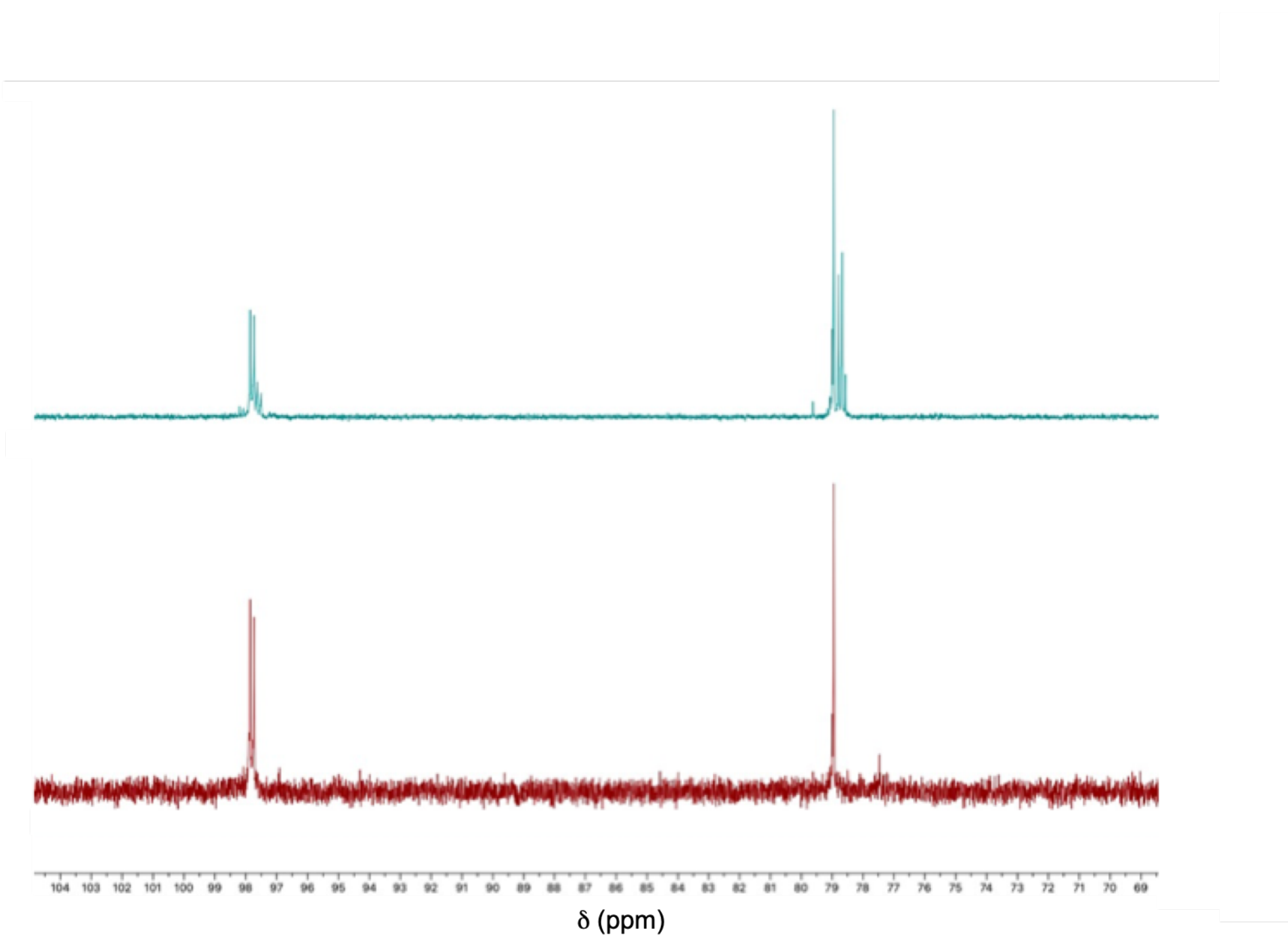

Figure S31: ^{11}B NMR, C_6D_6, 128 MHz, 298 K. Reactivity of reduction reaction mixture with $NaBPh_4$ (hexane soluble portion) (bottom) stacked with pre−cleaning (top).

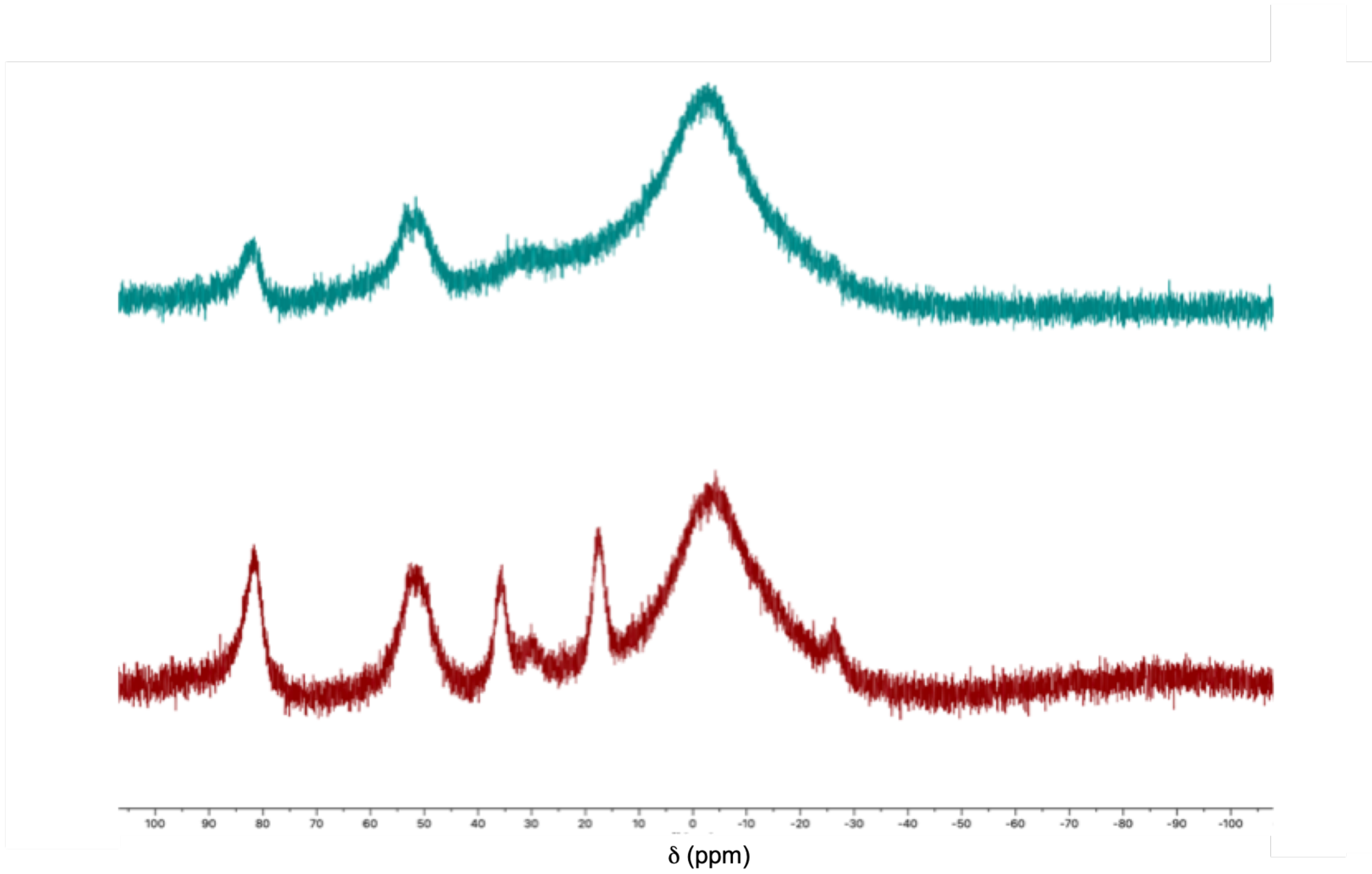

2. Mass spectrometry:

Figure S32: 2, LIFDI−HRMS: $C_{28}H_{55}ClFeO_2P_2Si$ ($[M]^+$) (604.2485, calculated); (604.2474, observed).

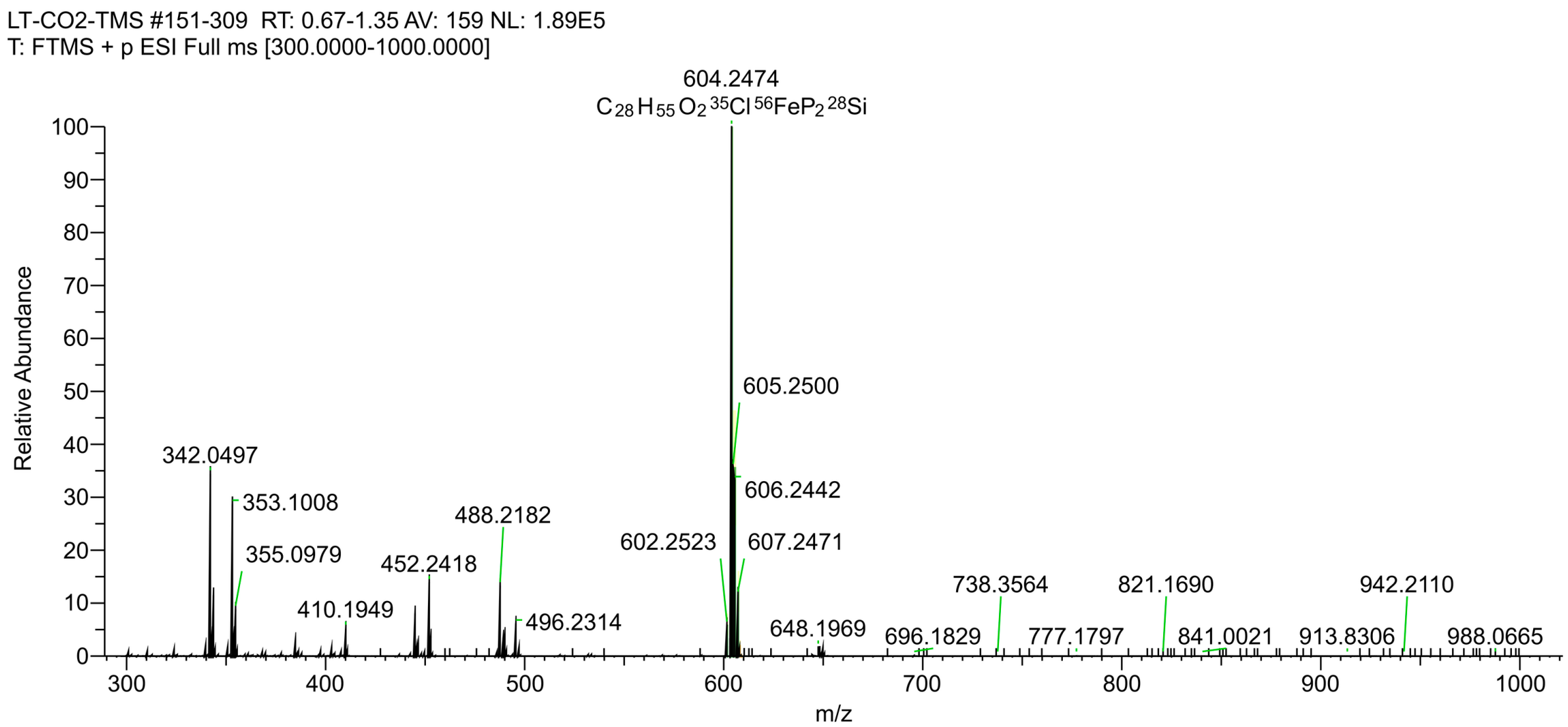

Figure S33: 2, LIFDI−HRMS: $C_{28}H_{55}ClFeO_2P_2Si$ ($[M]^+$) (604.2485, calculated); (604.2474, observed). Isotope pattern.

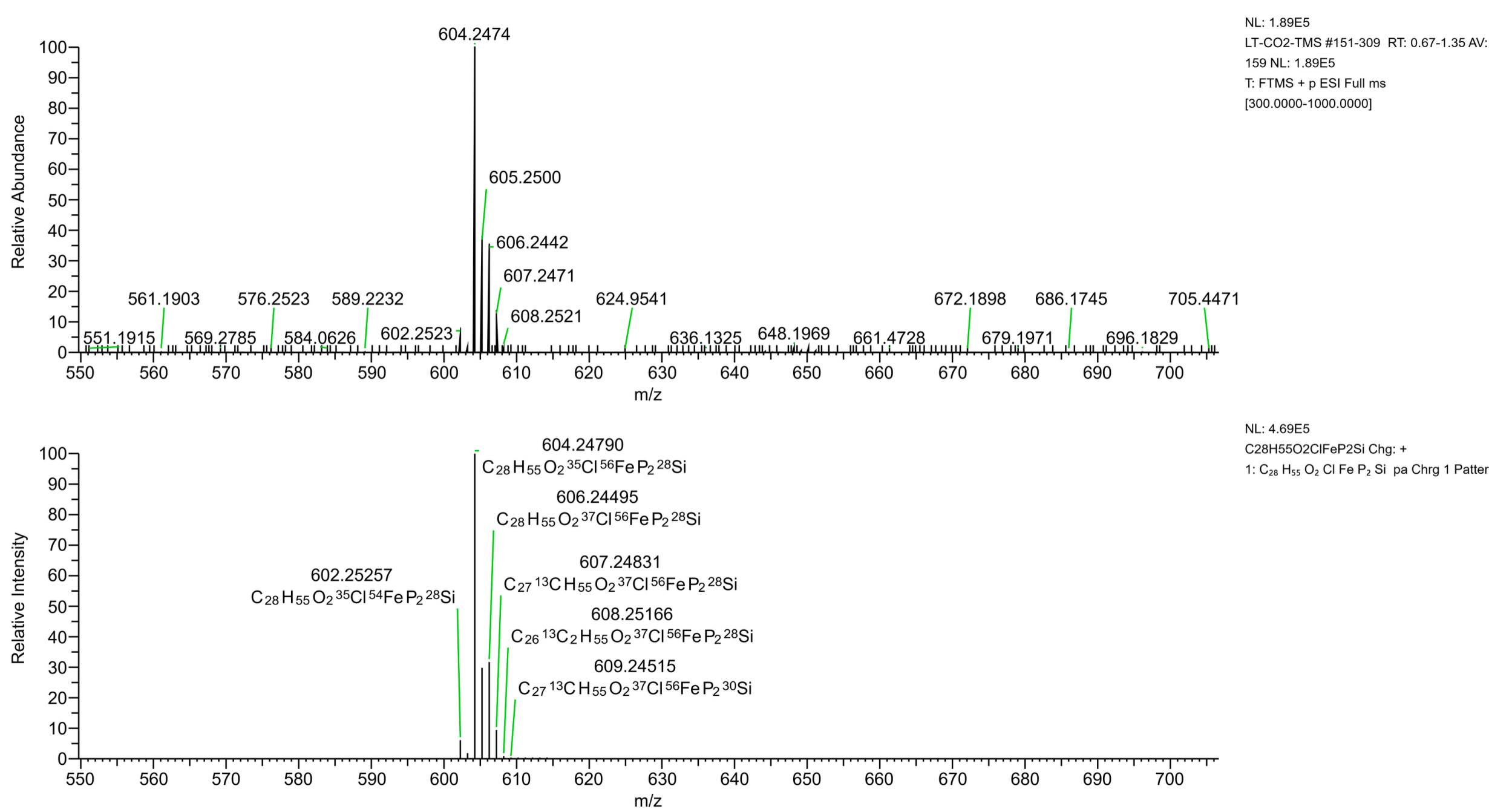

Figure S34: LIFDI−HRMS for reduction reaction mixture of **2** + 5 equiv. $HBCy_2$.

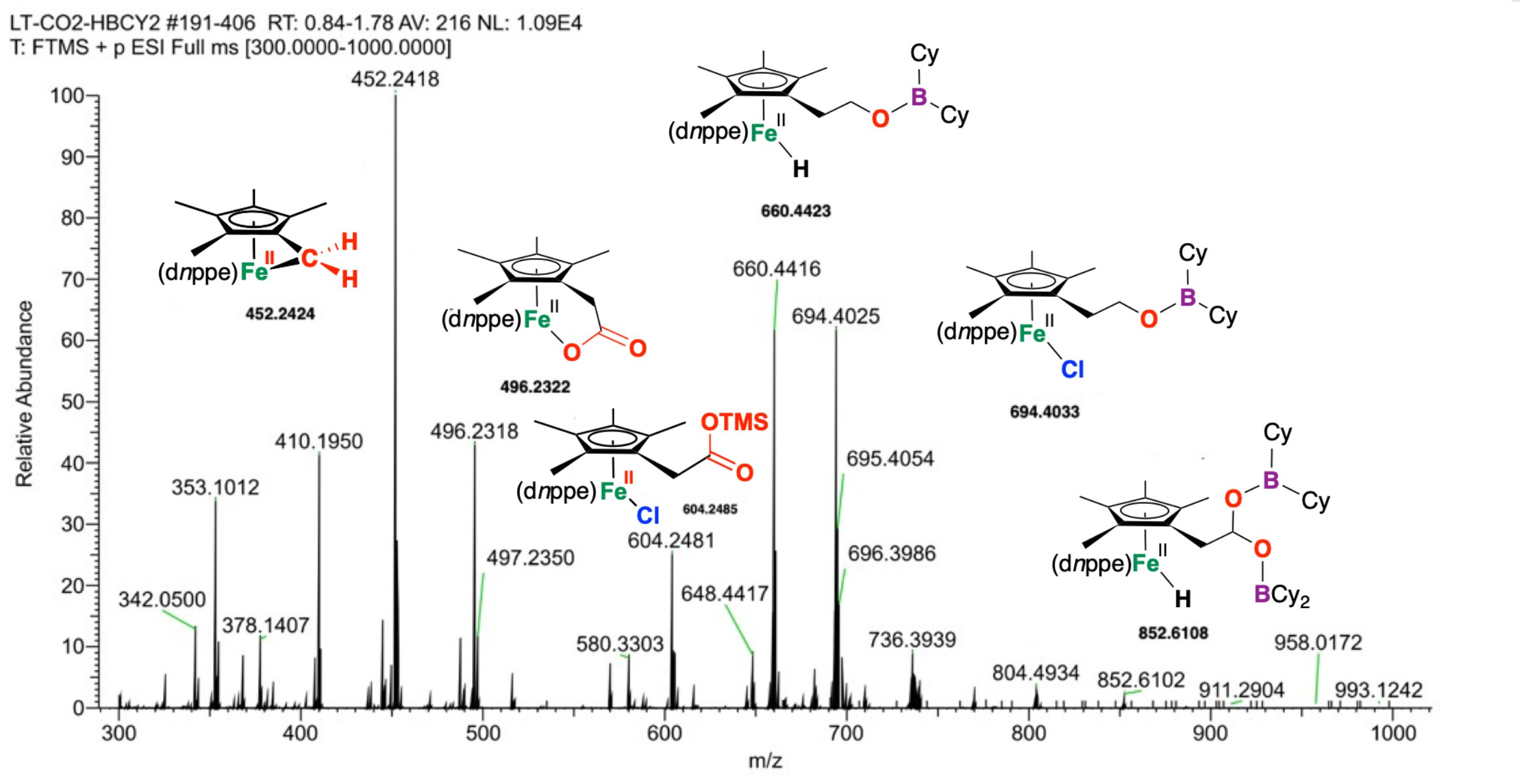

Figure S35: ESI(+)−MS [**5**−H]$^+$ $C_{25}H_{46}FeO_2P_2$ (496.232, calculated); (496.231, observed).

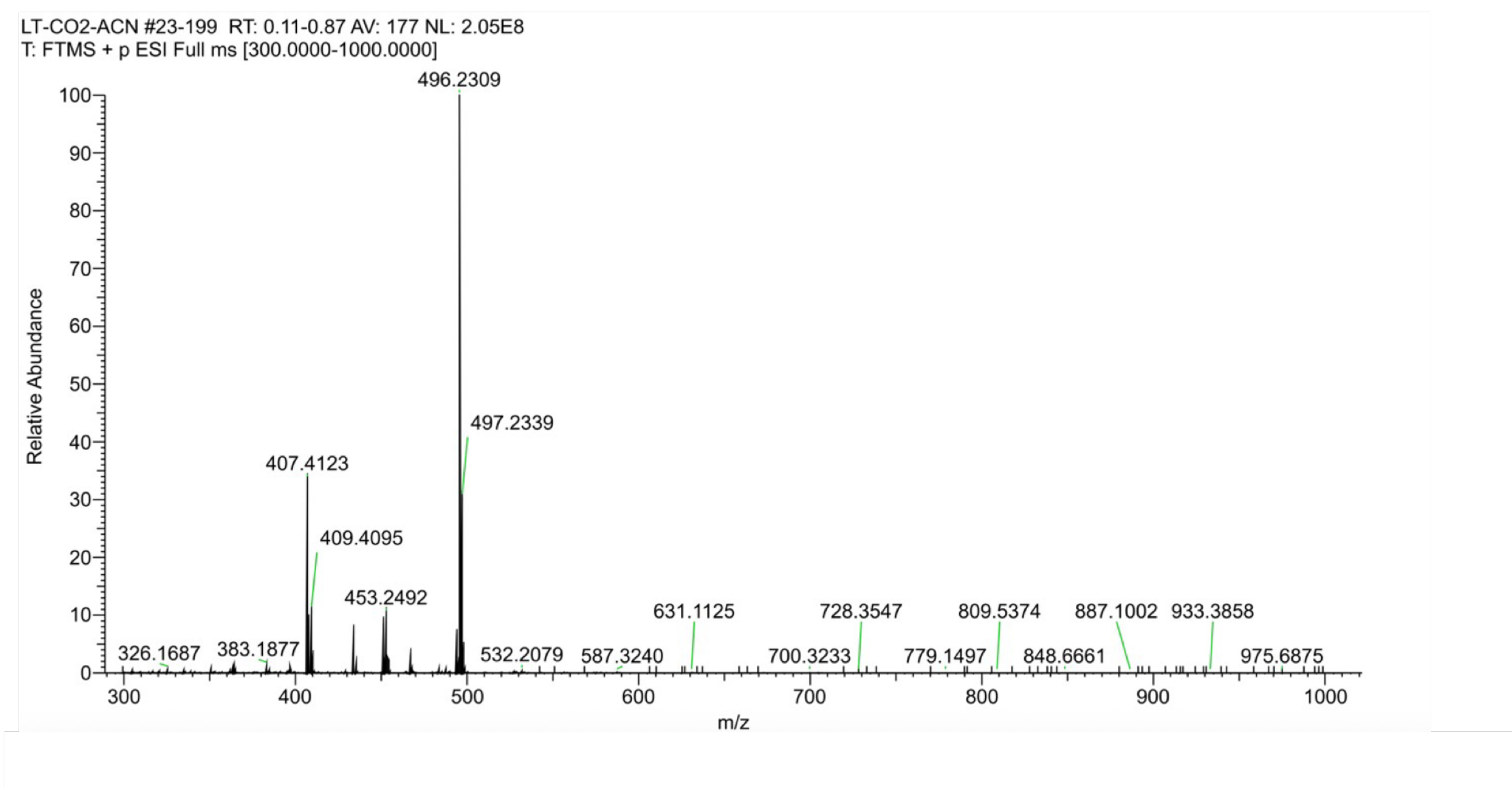

3. Crystallographic details:

CCDC **2575113** contains the supplementary crystallographic data for this paper. These data can be obtained free of charge from The Cambridge Crystallographic Data Centre *via* https://www.ccdc.cam.ac.uk.

Experimental for 2:

Crystal data were collected with Mo*K*α radiation on a Bruker Kappa Axis Apex2 diffractometer at Western University. All crystals were mounted on a MiTeGen loop with a small amount of Paratone N oil. Frame integration, scaling and absorption correction were performed using SAINT and SADABS integrated in Apex4 [1]. The structures were solved with the ShelXT [2] structure solution program using intrinsic phasing and refined with the ShelXL [3] refinement package using least squares on weighted F2 values for all reflections using OLEX2 [4].

Table S1. Crystallographic data for **2**.

Compound	**2**
Empirical Formula	$C_{28}H_{55}ClFeO_2P_2Si$
Formula Weight	605.05
Temperature/K	110.00
Crystal System	Monoclinic
Space group	$P2_1/c$
a/Å	15.276(6)
b/Å	13.058(5)
c/Å	16.903(6)
α/°	90
β/°	104.421(12)
γ/°	90
Volume/Å^3	3266(2)
Z	4
ρ_{calc}g/cm^3	1.231
μ/mm^{-1}	0.701
F(000)	1304.0
Crystal size/mm^3	0.53 x 0.195 x 0.111
Radiation	MoKα (λ = 0.71073)
2Θ range for data collection/°	5.938 to 60.986
Index ranges	$-21 \leq h \leq 21$, $-18 \leq k \leq 18$, $-24 \leq 1 \leq 22$
Reflections collected	160962
Independent reflections	9918 [R_{int} = 0.0981, R_{sigma} = 0.0417]
Data/restraints/parameters	9918/5/326
Goodness−of−fit on F^2	1.028
Final R indexes [I>=2σ (I)]	R_1 = 0.0430, wR_2 = 0.0958
Final R indexes [all data]	R_1 = 0.0775, wR_2 = 0.1158
Largest diff. peal/hole / e Å^3	0.72/−0.90

Where:

$R_1 = \Sigma \mid |F_o| - |F_c| \mid / \Sigma F_o$
$wR_2 = [\Sigma(w(F_o^2 - F_c^2)^2) / \Sigma(w F_o^4)]^{½}$
$GOF = [\Sigma(w(F_o^2 - F_c^2)^2) / (\text{No. of reflns.} - \text{No. of params.})]^{½}$

4. References:

(1) Bruker (2016). APEX3, SAINT and SADABS. Bruker AXS Inc., Madison, Wisconsin, USA.

(2) Sheldrick, G. M. *Acta Crystallogr. Sect. A* **2015**, *71* (1), 3–8. https://doi.org/10.1107/S2053273314026370.

(3) Sheldrick, G. M. *Acta Crystallogr. Sect. C* **2015**, *71* (1), 3–8. https://doi.org/10.1107/S2053229614024218.

(4) Dolomanov, O. V.; Bourhis, L. J.; Gildea, R. J.; Howard, J. A. K.; Puschmann, H. *J Appl. Crystallogr.* **2009**, *42* (2), 339–341. https://doi.org/10.1107/S0021889808042726.
