# Supplementary material for: CO2 silylation at a tucked-in iron(II) complex
Source: Beilstein J Org Chem. 2026 Sep 23;22:1295–302. doi: 10.3762/bjoc.22.104 (PMC13618351; doi:10.3762/bjoc.22.104)

checkCIF/PLATON report

Structure factors have been supplied for datablock(s) Complex_2

THIS REPORT IS FOR GUIDANCE ONLY. IF USED AS PART OF A REVIEW PROCEDURE FOR PUBLICATION, IT SHOULD NOT REPLACE THE EXPERTISE OF AN EXPERIENCED CRYSTALLOGRAPHIC REFEREE.

No syntax errors found.   CIF dictionary   Interpreting this report

Datablock: Complex_2

Bond precision:	C-C = 0.0033 A		Wavelength=0.71073
Cell:	a=15.276(6)	b=13.058(5)	c=16.903(6)
	alpha=90	beta=104.421(12)	gamma=90
Temperature:	110 K		

	Calculated	Reported
Volume	3266(2)	3266(2)
Space group	P 21/c	P 1 21/c 1
Hall group	-P 2ybc	-P 2ybc
Moiety formula	C28 H55 Cl Fe O2 P2 Si	C28 H55 Cl Fe O2 P2 Si
Sum formula	C28 H55 Cl Fe O2 P2 Si	C28 H55 Cl Fe O2 P2 Si
Mr	605.05	605.05
Dx,g cm-3	1.230	1.231
Z	4	4
Mu (mm-1)	0.700	0.701
F000	1304.0	1304.0
F000'	1307.43	
h,k,lmax	21,18,24	21,18,24
Nref	9967	9918
Tmin,Tmax	0.849,0.925	0.743,1.000
Tmin'	0.690	

Correction method= # Reported T Limits: Tmin=0.743 Tmax=1.000
AbsCorr = MULTI-SCAN

Data completeness= 0.995 Theta(max)= 30.493

R(reflections)= 0.0430(6889) wR2(reflections)= 0.1159(9918)

S = 1.029 Npar= 326

The following ALERTS were generated. Each ALERT has the format
test-name_ALERT_alert-type_alert-level.
Click on the hyperlinks for more details of the test.

Alert level C

```
PLAT905_ALERT_3_C Negative K value in the Analysis of Variance ...     -2.423 Report
PLAT910_ALERT_3_C Missing FCF Reflection(s) Below Theta(Min)[Deg]=       2.97 Note
                1  0  0,   1  1  0,   2  0  0,  -1  1  1,   0  1  1,   1  1  1,
               -1  0  2,   0  0  2,   0  1  2,
```

Alert level G

```
PLAT002_ALERT_2_G Number of Distance or Angle Restraints on AtSite          7 Note
PLAT171_ALERT_4_G The CIF-Embedded .res File Contains EADP Records          1 Report
PLAT176_ALERT_4_G The CIF-Embedded .res File Contains SADI Records          5 Report
PLAT191_ALERT_3_G A Non-default SADI Restraint Value has been used     0.0400 Report
PLAT191_ALERT_3_G A Non-default SADI Restraint Value has been used     0.0400 Report
PLAT232_ALERT_2_G Hirshfeld Test Diff (M-X)  Fe1      --Cl1      .        6.0 s.u.
PLAT232_ALERT_2_G Hirshfeld Test Diff (M-X)  Fe1      --P1       .        5.3 s.u.
PLAT232_ALERT_2_G Hirshfeld Test Diff (M-X)  Fe1      --P2       .        5.0 s.u.
PLAT301_ALERT_3_G Main Residue  Disorder .............(Resd    1)          9% Note
PLAT410_ALERT_2_G Short Intra H...H Contact  H24B     ..H27A     .       1.96 Ang.
                                                      x,y,z  =      1_555 Check
PLAT412_ALERT_2_G Short Intra XH3 .. XHn     H10A     ..H26A     .       2.04 Ang.
                                                      x,y,z  =      1_555 Check
PLAT773_ALERT_2_G Check long C-C Bond in CIF: C27B     --C28B            1.72 Ang.
PLAT860_ALERT_3_G Number of Least-Squares Restraints ............           5 Note
PLAT883_ALERT_1_G Absent Datum for _atom_sites_solution_primary ..     Please Do !
PLAT912_ALERT_4_G Missing # of FCF Reflections Above STh/L=  0.600         40 Note
PLAT933_ALERT_2_G Number of HKL-OMIT Records in Embedded .res File          8 Note
               -1  1  1,   0  0  2,   0  1  1,   0  1  2,   1  0  0,   1  1  0,
                1  1  1,   2  0  0,
PLAT969_ALERT_5_G The 'Henn et al.' R-Factor-gap value ..........       2.982 Note
              Predicted wR2: Based on SigI**2  3.89 or SHELX Weight 11.26 Note
PLAT978_ALERT_2_G Number C-C Bonds with Positive Residual Density.          7 Info
```

---

0 **ALERT level A** = Most likely a serious problem - resolve or explain
0 **ALERT level B** = A potentially serious problem, consider carefully
2 **ALERT level C** = Check. Ensure it is not caused by an omission or oversight
18 **ALERT level G** = General information/check it is not something unexpected

1 ALERT type 1 CIF construction/syntax error, inconsistent or missing data
9 ALERT type 2 Indicator that the structure model may be wrong or deficient
6 ALERT type 3 Indicator that the structure quality may be low
3 ALERT type 4 Improvement, methodology, query or suggestion
1 ALERT type 5 Informative message, check

Validation response form

Please find below a validation response form (VRF) that can be filled in and pasted into your CIF.

```
# start Validation Reply Form
_vrf_PLAT905_Complex_2
;
PROBLEM: Negative K value in the Analysis of Variance ...     -2.423 Report
RESPONSE: ...
;
_vrf_PLAT910_Complex_2
;
PROBLEM: Missing FCF Reflection(s) Below Theta(Min)[Deg]=       2.97 Note
RESPONSE: ...
;
# end Validation Reply Form
```

It is advisable to attempt to resolve as many as possible of the alerts in all categories. Often the minor alerts point to easily fixed oversights, errors and omissions in your CIF or refinement strategy, so attention to these fine details can be worthwhile. It is up to the individual to critically assess their own results and, if necessary, seek expert advice.

PLATON version of 23/04/2026; check.def file version of 30/03/2026

duplicate check

No duplication found

Datablock Complex_2 - ellipsoid plot

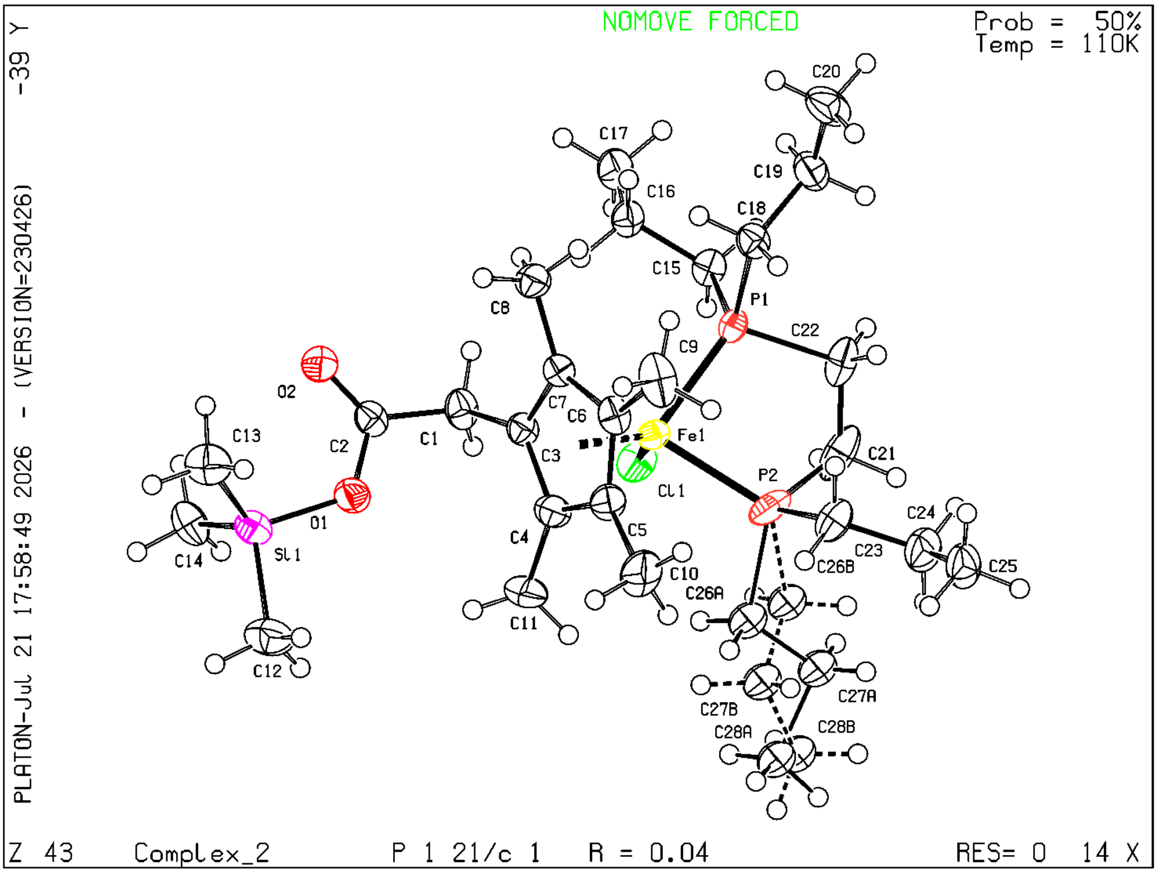

Supplement: Supplementary file 2 [file Beilstein_J_Org_Chem-22-1295-s002.zip › CO2_Silylation_2026_CheckCif_BJOC.pdf]
